# Systemic inflammation and metabolic disturbances underlie inpatient mortality among ill children with severe malnutrition

**DOI:** 10.1126/sciadv.abj6779

**Published:** 2022-02-16

**Authors:** Bijun Wen, James M. Njunge, Celine Bourdon, Gerard Bryan Gonzales, Bonface M. Gichuki, Dorothy Lee, David S. Wishart, Moses Ngari, Emmanuel Chimwezi, Johnstone Thitiri, Laura Mwalekwa, Wieger Voskuijl, James A. Berkley, Robert HJ Bandsma

**Affiliations:** 1 Department of Nutritional Sciences, Faculty of Medicine, University of Toronto, Toronto, Canada.; 2 Department of Translational medicine, Hospital for Sick Children, Toronto, Canada.; 3 The Childhood Acute Illness & Nutrition Network, Nairobi, Kenya.; 4 KEMRI/Wellcome Trust Research Programme, Kilifi, Kenya.; 5 Nutrition, Metabolism and Genomics Group, Division of Human Nutrition and Health, Wageningen University & Research, Wageningen, Netherlands.; 6 The Metabolomics Innovation Centre, Edmonton, Alberta, Canada.; 7 Department of Paediatrics, Coast General Hospital, Mombasa, Kenya.; 8 Department of Global Health, Amsterdam Institute for Global Health and Development, Amsterdam University Medical Centres, Amsterdam, Netherlands.; 9 Department of Pediatrics, the College of Medicine, University of Malawi, Blantyre, Malawi.; 10 Centre for Tropical Medicine & Global Health, Nuffield Department of Medicine, University of Oxford, Oxford, UK.; 11 Department of Biomedical Sciences, the College of Medicine, University of Malawi, Blantyre, Malawi.

## Abstract

Children admitted to hospital with an acute illness and concurrent severe malnutrition [complicated severe malnutrition (CSM)] have a high risk of dying. The biological processes underlying their mortality are poorly understood. In this case-control study nested within a multicenter randomized controlled trial among children with CSM in Kenya and Malawi, we found that blood metabolomic and proteomic profiles robustly differentiated children who died (*n* = 92) from those who survived (*n* = 92). Fatalities were characterized by increased energetic substrates (tricarboxylic acid cycle metabolites), microbial metabolites (e.g., propionate and isobutyrate), acute phase proteins (e.g., calprotectin and C-reactive protein), and inflammatory markers (e.g., interleukin-8 and tumor necrosis factor–α). These perturbations indicated disruptions in mitochondria-related bioenergetic pathways and sepsis-like responses. This study identified specific biomolecular disturbances associated with CSM mortality, revealing that systemic inflammation and bioenergetic deficits are targetable pathophysiological processes for improving survival of this vulnerable population.

## Teaser

A sepsis-like omic profile underlies death among sick malnourished children, altering our thinking on management strategies.

## INTRODUCTION

Severe malnutrition is a public health problem affecting over 16 million children in low- and middle-income countries (*1*). It is associated with increased risk of mortality due to infectious diseases, including pneumonia and diarrhea, and accounts for nearly half of global deaths in children under 5 years (*1*). Complicated severe malnutrition (CSM) is defined as having one or more Integrated Management of Childhood Illness clinical danger signs, evidence of infection, severe edema, or unable to sufficiently feed, in addition to being severely malnourished (*2*). Children with CSM are primarily admitted to hospital because of acute illnesses, including serious infections, and are often only identified as severely malnourished at that time. The World Health Organization (WHO) guidelines for management of children with severe malnutrition recommend empiric antibiotics, treatment of other medical conditions, and correction of nutritional deficiencies through therapeutic feeding (*2*).

Despite following WHO guidelines, reported inpatient mortality of children with CSM ranges 8 to 25% in African hospitals (*3*). Characteristics associated with increased risk of death, including HIV, young age, and very low anthropometry, are relevant for clinical management but do not indicate specific pathophysiological mechanisms that could be therapeutically targeted.

CSM is associated with substantial disturbances in metabolism, including altered protein and lipid metabolism, and increased susceptibility to dysglycemia (*4*–*7*). Comparing metabolic profiles of sick children with CSM before and after medical and nutritional stabilization, Bartz *et al.* (*4*) demonstrated that the metabolome at admission is characterized by high circulating levels of nonesterified fatty acids, ketones, even-chain acylcarnitines, and low levels of amino acids. Derangements in specific amino acids, acylcarnitines, and phospholipids corroborated studies comparing CSM to nonmalnourished hospitalized children or community controls (*5*, *6*). Limited data from children and animal models indicate that these disturbances could be attributed to impairments in nutrient utilization and mitochondrial function (*7*–*11*). Mitochondria are bioenergetic organelles responsible for β-oxidation, tricarboxylic acid (TCA) cycle, and oxidative phosphorylation, which are vital processes of converting nutrients into ATP to support normal cellular functions. In a malnutrition rat model, malnutrition alone impairs mitochondrial function, reducing the capacity to oxidize free fatty acids (*11*).

Although disturbed energy metabolism has been described, its relationship with survival among children with CSM has not been explicitly demonstrated. To our knowledge, only two small studies have reported biomolecular perturbations in CSM related to death (*4*, *12*). Bartz *et al.* (*4*) reported that low levels of leptin at admission were a predictor of subsequent inpatient mortality. However, except for leptin, other biomolecular markers quantified in this study were not associated with mortality when adjusted for confounders. Circulating leptin reflects fat mass reserve with immune regulatory functions; it is unclear whether the observed difference was related to differences in body composition or recent food intake between survivors and nonsurvivors. Another small study from our own group demonstrated that increased systemic inflammatory markers were associated with inpatient mortality among children with CSM (*12*).

A hallmark of infection is stimulation of host catabolism and activation of inflammatory responses, which are nutritionally costly bioenergetic processes (*13*). Dysregulation in host immune responses can lead to a sepsis syndrome, a life-threatening condition. A recent meta-analysis among well-nourished populations from high-resource countries identified that sepsis nonsurvivors had impaired energy metabolism, uncontrolled inflammation, proteolysis, and defects in organ healing (*14*). However, in the context of CSM, the relationship between host response to infection and mortality has not been characterized.

We postulated that the interplay between metabolic disturbances in energy metabolism and overwhelming host response to infection are central drivers of inpatient mortality in childhood CSM. To examine this hypothesis, we characterized biological processes associated with inpatient mortality among children with CSM using a multiomics case-control approach.

## RESULTS

### Characteristics of participants

Participants’ characteristics at enrolment are detailed in Table 1. Children included in this matched case-control study had a median age of 15 months [interquartile range (IQR): 9 to 27] and 45% were female. The median time to death was 6 days (IQR: 4 to 9), and time to discharge was 8 days (IQR: 6 to 11) after hospital admission. Anthropometric, demographic, and clinical characteristics were similar between nonsurvivors (NS) and survivors (S). Besides being severely wasted, most children were severely stunted. The prevalence of most clinical features was similar between groups, although nonsurvivors more often had lower chest indrawing (*P* < 0.001) and diarrhea (*P* = 0.02). Participants in this nested study were representative of children from the F75 trial.

**Table 1.** Characteristics of study participants.

	**Trial participants**			**Nested case-control participants**		
	**Nonsurvivors** **(*n* = 127)**	**Survivors (*n* = 653)**	***P***	**Nonsurvivors** **(NS; *n* = 92)**	**Survivors** **(S; *n* = 92)**	***P***
Age (months)†, means ± SDs	21.4 ± 16.6	22.7 ± 18.7	0.45	21.3 ± 17.7	21.2 ± 15.3	0.95
Gender (female), (%)	64 (50.4%)	296 (45.3%)	0.30	42 (45.7%)	42 (45.7%)	1.00
**Anthropometric and nutritional features**						
MUAC (cm)†, means ± SD	10.7 ± 1.6	11.3 ± 1.4	<0.001 *	10.7 ± 1.6	10.9 ± 1.4	0.41
WHZ, means ± SD	−3.8 ± 1.5 (*n* = 120)	−3.2 ± 1.5 (*n* = 617)	<0.001 *	−3.8 ± 1.6 (*n* = 86)	−3.4 ± 1.6 (*n* = 90)	0.16
WAZ, means ± SD	−4.3 ± 1.4	651; −3.9 ± 1.4	<0.001 *	−4.4 ± 1.4	−4.2 ± 1.4	0.28
HAZ, means ± SD	−3.3 ± 1.7 (*n* = 124)	−3.0 ± 1.7 (*n* = 652)	0.07	−3.4 ± 1.8 (*n* = 89)	−3.3 ± 1.6	0.76
BMIZ, means ± SD	−3.2 ± 0.9 (*n* = 4)	−3.3 ± 2.1 (*n* = 34)	0.96	−3.3 ± 1.1 (*n* = 3)	−4.2 ± 0.1 (*n* = 2)	0.33
Nutritional edema present, *n* (%)	47 (37.0%)	199 (*n* = 650, 30.6%)	0.16	29 (31.5%)	26 (28.3%)	0.63
**Clinical features**						
HIV antibody test†, *n* (%)						
Positive	47 (37.0%)	122 (18.7%)		35 (38.0%)	36 (39.1%)	
Refused/died before testing	16 (12.6%)	24 (3.7%)	<0.001 *	12 (13.0%)	12 (13.0%)	0.99
Diarrhea, *n* (%)	61 (48.0%)	267 (40.9%)	0.14	45 (48.9%)	30 (32.6%)	0.02 *
Severe pneumonia, *n* (%)	40 (31.5%)	153 (23.4%)	0.05	33 (35.9%)	22 (23.9%)	0.08
Chest indrawing	39 (30.7%)	105 (16.1%)	<0.001 *	33 (35.9%)	13 (14.1%)	<0.001 *
Fever, *n* (%)	36 (28.3%)	180 (27.6%)	0.86	31 (33.7%)	22 (23.9%)	0.14
Vomiting, *n* (%)	32 (25.2%)	183 (28.0%)	0.52	26 (28.3%)	18 (19.6%)	0.17
Impaired consciousness, *n* (%)	10 (7.9%)	18 (2.8%)	0.01 *	5 (5.4%)	2 (2.2%)	0.25
Cerebral palsy, *n* (%)	17 (13.4%)	99 (15.2%)	0.61	12 (13.0%)	15 (16.3%)	0.54
Chronic cough, *n* (%)	6 (4.7%)	44 (6.7%)	0.40	5 (5.4%)	10 (10.9%)	0.18
Hypothermia, *n* (%)	8 (6.3%)	35 (5.4%)	0.67	6 (6.5%)	8 (8.7%)	0.58
Convulsions, *n* (%)	7 (5.5%)	30 (4.6%)	0.66	6 (6.5%)	1 (1.1%)	0.05
Malaria, *n* (%)	6 (4.7%)	57 (8.7%)	0.13	4 (4.3%)	5 (5.4%)	0.73
Tuberculosis, *n* (%)	4 (3.1%)	12 (1.8%)	0.34	3 (3.3%)	3 (3.3%)	1.00
Anemia, *n* (%)	4 (3.1%)	22 (3.4%)	0.90	4 (4.3%)	2 (2.2%)	0.41
**Original trial features**						
Recruitment site, *n* (%)						
Kilifi County Hospital	23 (18.1%)	156 (23.9%)		23 (25.0%)	21 (22.8%)	
Coast Provincial General Hospital	40 (31.5%)	250 (38.3%)		36 (39.1%)	43 (46.7%)	
Queen Elizabeth Central Hospital	64 (50.4%)	247 (37.8%)	0.03 *	33 (35.9%)	28 (30.4%)	0.58
Randomization arm (modified F75), *n* (%)	68 (53.5%)	322 (49.3%)	0.38	50 (54.3%)	41 (44.6%)	0.19

†Factors used in propensity score matching of case-controls. *P < 0.05.

### Metabolomic derangements at admission are associated with mortality

**Fig. 1.**
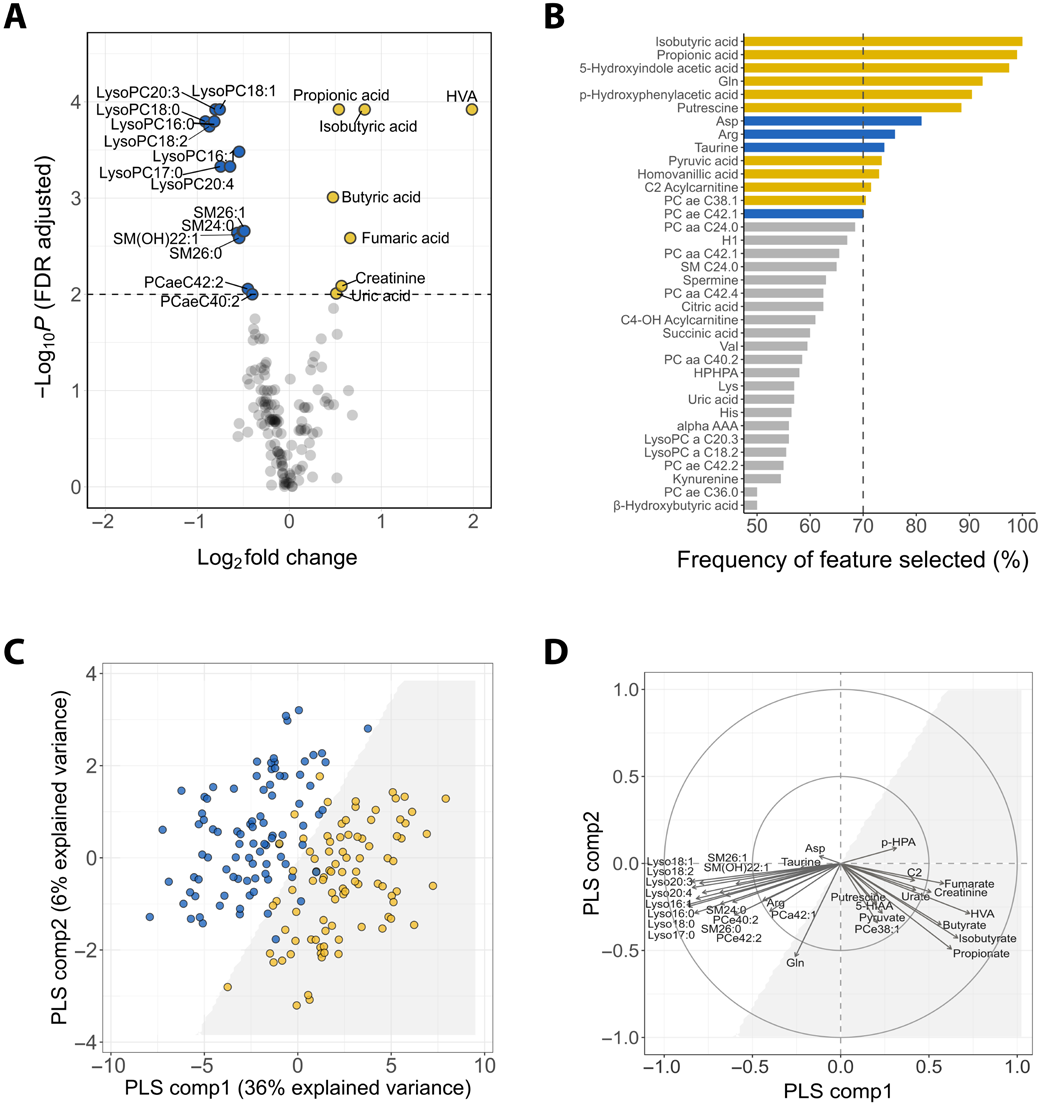
Metabolomic signatures associated with mortality. (**A**) Volcano plot of 144 measurable metabolites. Top significant metabolites between NS and S are above the dashed line (*P*_FDR_ < 0.01). Yellow and blue circles correspond to elevated and reduced levels in NS compared to S, respectively. (**B**) Frequency of metabolite selection by the elastic net multivariate analysis. Bars correspond to the percentage of the respective metabolite being selected by the model out of the 200 bootstrap samples. Influential metabolites are those selected >70% (dashed line) of times, with bar color denoting mean concentration higher (yellow) or lower (blue) in NS compared to S. (**C**) Score plot of individuals (NS in yellow; S in blue) clustered by multilevel partial least square discriminant analysis (PLS-DA) of 32 differential metabolites. The interface between the white and the gray shaded area represents the classification decision line. The first two components explained 42% of the total variance. Model performance and validity measures were as follows: cross-validated AUC = 0.96 ± 0.006, misclassification rate = 0.12 ± 0.01, DR^2^ = 0.74 ± 0.001 and DQ^2^ = 0.63 ± 0.02. (**D**) Correlations between the 32 differential metabolites and PLS components. Arrows denote the direction and magnitude of correlations. See fig. S2 for boxplots of concentrations of the top contributing metabolites (correlation strength > 0.5). HVA, homovanillic acid; 5-HIAA, 5-hydroxyindole acetate; p-HPA, *p*-hydroxyphenylacetate; C2, acetylcarnitine.

Of the 206 targeted metabolites, 144 met quality control criteria and were retained for analysis (table S1). Univariate analysis identified 21 metabolites as the top significant analytes between NS and S at *P*_FDR_ < 0.01 (Fig. 1A and table S2). In multivariable analysis, 14 metabolites were selected by elastic net as influential analytes (Fig. 1B). For completeness, we considered the union of the top significant analytes (*P*_FDR_ < 0.01) and the multivariable influential analytes as differential analytes, yielding a total 32 differential metabolites (fig. S1A). For matched case-control pairs, these 32 differential metabolites confidently discriminated NS from S [area under the receiver-operating characteristic curve (AUC) = 0.96, 95%CI: (0.95 to 0.96); Fig. 1C]. The admission metabolome of NS was predominantly characterized by elevated levels of organic acids [isobutyrate, propionate, butyrate, homovanillate (HVA), fumarate, urate, and pyruvate], creatinine, and acetylcarnitine (C2), and reduced levels of 14 long-chain lysophospholipid (lysoPC), sphingolipid (SM), and phospholipid (PC) species (lysoPC a C16:0, lysoPC a C16:1, lysoPC a C17:0, lysoPC a C18:0, lysoPC a C18:1, lysoPC a C18:2, lysoPC a C20:3, lysoPC a C20:4, SM C24:0, SM C26:0, SM C26:1, SM(OH)C22:1, PCaaC42:2, and PCaeC40:2), as shown by the partial least squares (PLS)-correlation plot (Fig. 1D). These data indicate that increased levels of specific organic acids together with depletion of long-chain lipids were associated with mortality. Boxplots comparing concentrations of these metabolites between groups and also healthy community subjects are shown in fig. S2. Consistent findings were found with edema or weight-for-height *Z*-score (WHZ) adjusted (fig. S3).

### Altered energy metabolism is associated with mortality

**Fig. 2.**
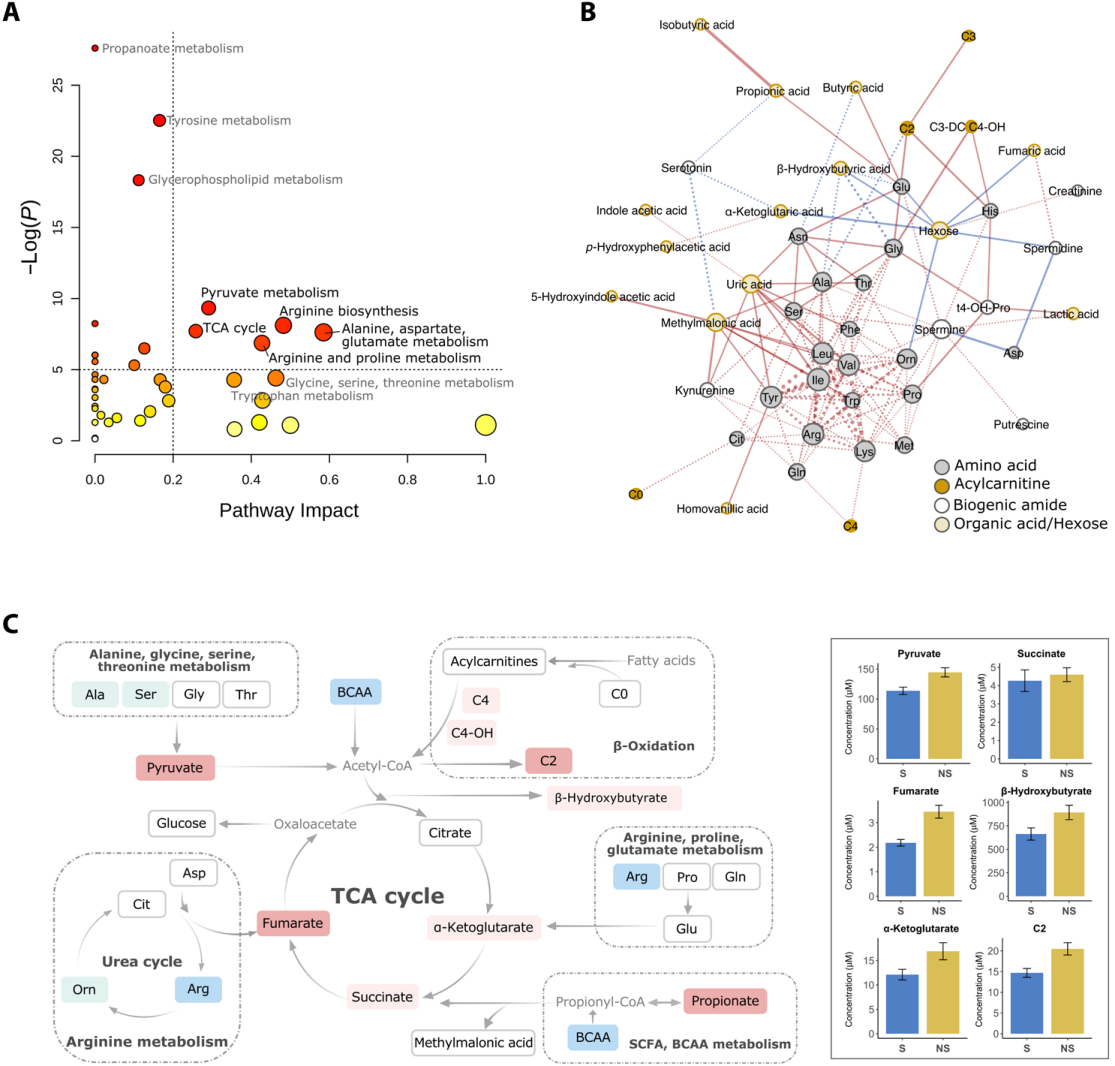
Metabolic pathways associated with mortality. (**A**) Pathways associated with metabolomic alterations in NS. Pathway impact indicates the sum of importance of the altered metabolites in the impacted pathway based on pathway topology; the −log(*P*) are test statistics for quantitative pathway enrichment analysis based on concentration differences between groups. Impacted pathways are above the dashed lines [impact >0.2 and −log(*P*) >5]. (**B**) Differential correlation network of metabolites. Nodes are metabolites, and edges connect metabolite pairs with differential correlations between groups (*P*_∆r_ < 0.05). Solid edges denote increased and dashed edges denote reduced strengths of correlation in NS. The relative width of edges corresponds to the absolute difference in correlation strengths. Since no reversed correlation was detected, positive correlations are indicated by red and negative correlations are indicated by blue edges. Node color and size correspond to metabolite classes and node degree, respectively. (**C**) Mapping of univariately altered metabolites onto impacted pathways. Metabolites boxed in dark red or blue rectangles were significantly increased or decreased in NS (*P*_FDR_ < 0.05), respectively; those in light red or blue showed marginal increase or decrease in NS (*P*_FDR_ < 0.1); those boxed in white were comparable between groups. Grayed out metabolites were not measured. Right panel listed concentrations of key TCA cycle metabolites.

Among 144 measured metabolites, 103 were annotated in the Kyoto Encyclopedia of Genes and Genomes (KEGG) database and were used for pathway analysis (table S1). Nonannotated metabolites were predominantly phospholipids as expected. Five metabolic pathways were most strongly affected by the differential metabolomics [pathway impact > 0.2 and −log(*P*) > 5], including (i) pyruvate metabolism; (ii) arginine biosynthesis; (iii) the TCA cycle pathway; (iv) alanine, asparate, and glutamate metabolism; and (v) arginine and proline metabolism (Fig. 2A). Moreover, levels of several metabolite ratios were differentially affected. Fischer’s ratio [branched chain amino acids (BCAA)/tyrosine and phenylalanine], which has been used as an indicator of liver metabolic function, and total urea cycle amino acids (citrulline, ornithine, arginine, and aspartate) were significantly lower in NS (Fischer’s fold change = 0.8, *P*_FDR_ = 0.02; urea cycle fold change = 0.8, *P*_FDR_ = 0.03; fig. S2). The ratios of acetylcarnitine to carnitine (C2/C0) that mark β-oxidation activity and those of kynurenine to tryptophan that indicate systemic immune activation were significantly elevated among NS (C2/C0 fold change = 1.6, *P*_FDR_ = 0.01; kynurenine/tryptophan fold change = 1.4, *P*_FDR_ = 0.04; fig. S2).

If the underlying state of the metabolic system were different between NS and S, in addition to changes in average metabolite abundances, the correlation patterns among metabolites would expectedly be differentially affected, whether the correlations become stronger or weaker (*15*). Therefore, we compared metabolite-metabolite correlation patterns between NS and S, focusing on amino acids, biogenic amides, acylcarnitines, and organic acids, given their central roles in nutrient oxidation. A differential correlation network was revealed (Fig. 2B), which illustrates global differences in metabolism between NS and S. In this network, positively intercorrelated amino acids densely clustered together. Among NS, these correlations were markedly weakened (dashed edges), with BCAA, arginine, and tyrosine showing the highest number of differential correlations (high node degrees), substantiating perturbations in the metabolism among these amino acids. For example, the positive correlations between amino acids in the urea cycle observed in S were significantly weakened in NS (arginine-ornithine: *r*_S_ = 0.7, *r*_NS_ = 0.1, and *P*_∆r_ < 0.0001; citrulline-arginine: *r*_S_ = 0.4, *r*_NS_ = 0.04, and *P*_∆r_ = 0.03). It is worth noting that meaningful changes in metabolite-metabolite correlations are not necessarily confined to adjacent metabolites in a pathway or metabolites with mean concentration changes (*15*). Tyrosine is upstream of dopamine biosynthesis, whereas HVA is an end product of dopamine degradation. Increased positive correlation of tyrosine with HVA in NS compared to S (*P*_∆r_ = 0.02) may suggest differential regulation of this pathway (fig. S4). Moreover, although the average concentration of hexoses did not differ between NS and S, the correlations between hexoses and TCA metabolites (α-ketoglutarate, β-hydroxybutyrate, and fumarate) were altered. In particular, hexoses were tightly maintained at a constant level irrespective of TCA metabolite levels in S, whereas hexoses were negatively correlated with TCA metabolite levels in NS (fig. S4).

Overall, NS exhibited changes in mean concentrations and underlying correlation patterns of metabolites involved in specific amino acids and central energy utilization pathways. A schematic map of death-related metabolic alterations overlaid on the impacted pathways is shown in Fig. 2C. It can be noted that, in NS, reductions of amino acids were accompanied by accumulations of downstream catabolic products (i.e., TCA cycle metabolites), implying obstructions in complete nutrient oxidation.

### Acute phase and inflammatory responses are associated with mortality

**Fig. 3.**
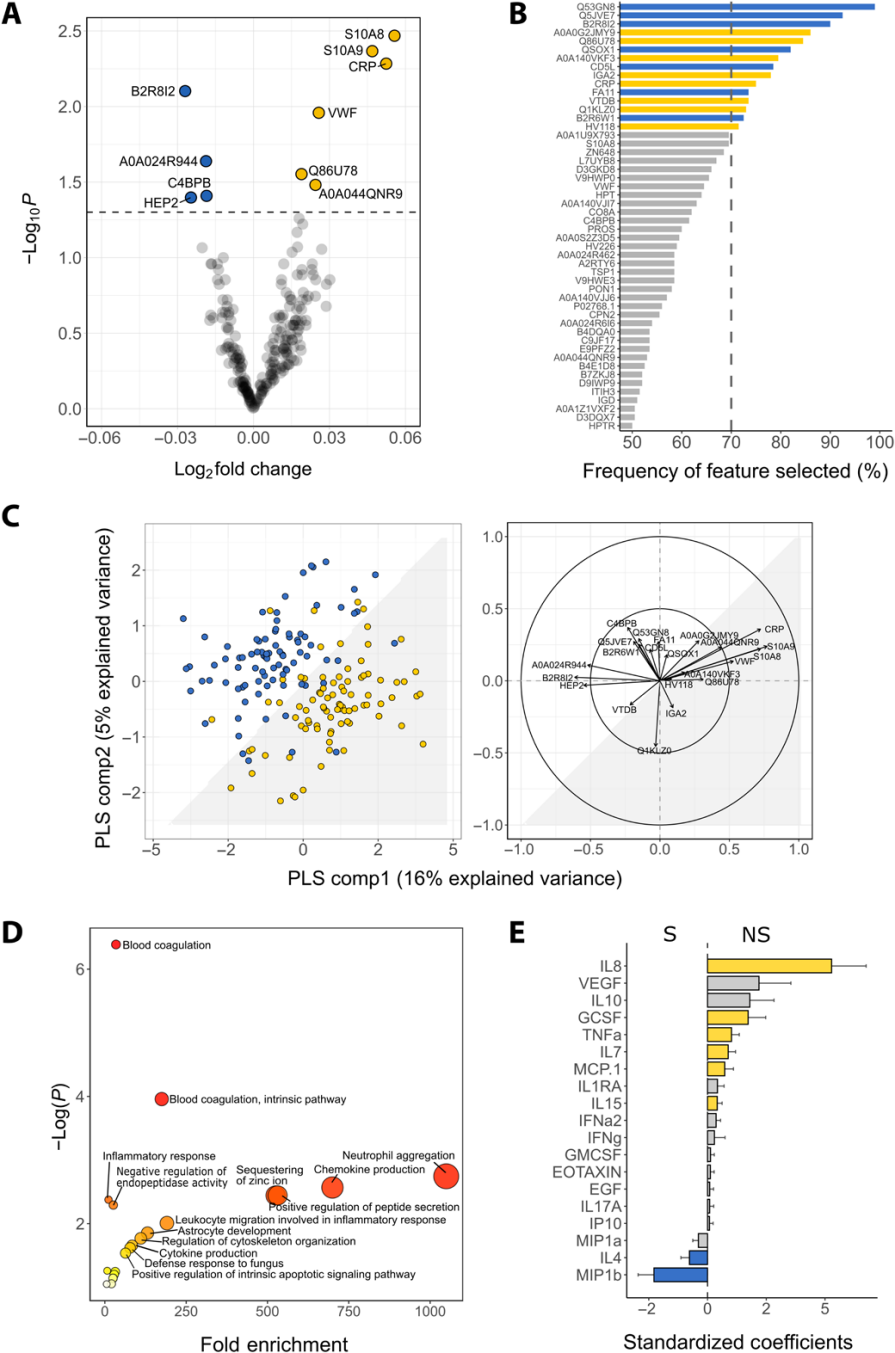
Proteins and biological processes associated with mortality. (**A**) Volcano plot of 229 quantified proteins. Significant differential proteins are above the dashed line (*P* < 0.05). Yellow and blue circles correspond to elevated and reduced levels in NS compared to S, respectively. (**B**) Frequency of influential proteins being selected by the elastic net multivariate analysis. Bars correspond to the percentage of the respective metabolite being selected by the model out of the 200 bootstrap samples. Influential proteins are those selected >70% (dashed line) of times, with bar color denoting mean concentration higher (yellow) or lower (blue) in NS compared to S. (**C**) Left: score plot of individuals (NS in yellow; S in blue) clustered by multilevel PLS-DA of 22 differential proteins. The interface between the white and the gray shaded area represents the classification decision line. The first two components explained 21% of the total variance of the 22 differential proteins. Model performance and validity measures were as follows: AUC = 0.86 ± 0.019, misclassification rate = 0.19 ± 0.024, DR^2^ = 0.50 ± 0.02 and DQ^2^ = 0.37 ± 0.04. Right: Correlations between the 22 differential proteins and PLS components. Arrows denote direction and magnitude of correlations. (**D**) Biological processes enrichment analysis of up-regulated proteins among NS associated with mortality. *P* value depicts the probability that a particular biological process is enriched in a group of proteins relative to other biological processes. (**E**) Standardized coefficients of inflammatory mediators. Color-filled bars represent significant cytokines associations with death (*P* < 0.05), with yellow denoting increase and blue denoting decrease in concentration in NS compared to S.

We next compared the levels of 229 quantified untargeted proteins between NS and S. In univariate analysis, 10 proteins had altered levels between NS and S (Fig. 3A). Among NS, subunits of calprotectin (S10A8/S10A9), C-reactive protein (CRP), von Willebrand factor (vWF), and angiotensinogen (AGT; Q86U78) were increased, while histidine-rich glycoprotein (HRG; B2R8I2), complement component 4 binding protein (C4BPB), antithrombin (Serpin C1), and heparin cofactor 2 (Serpin D1) were decreased (table S3). In multivariable elastic net analysis, 15 proteins were selected as influential analytes in distinguishing NS from S including CRP, AGT, and HRG proteins identified by the univariate analysis (Fig. 3B). Other proteins selected included coagulation factor X (Q5JVE7), macrophage-stimulating 1 (Q53GN8), coagulation factor XI (FA11), vitamin D-binding–protein (VTDB), quiescin sulfhydryl oxidase 1 (QSOX1), and immunoglobulins. Discriminant performance of these 22 differential analytes yielded an AUC of 0.86 [95% confidence interval (CI): (0.86 to 0.87)] (Fig. 3C). Consistent findings were found with edema or WHZ adjusted (fig. S3).

Biological process enrichment analysis indicated that several processes were positively linked to mortality, including coagulation, neutrophil aggregation, chemokine and cytokine production, positive regulation of peptide secretion, and defense responses to fungi, among others (Fig. 3D). Since we had hypothesized that systemic inflammation could be related to inpatient mortality in children with CSM, we quantified chemokines and cytokines involved in inflammation in plasma. Eight inflammatory mediators distinguished NS and S (*P* < 0.05; Fig. 3E). Specifically, NS had significantly higher levels of proinflammatory cytokines tumor necrosis factor–α (TNFα), interleukin-7 (IL7), IL8, IL15, granulocyte colony-stimulating factor (GCSF), and chemokine (C-C motif) ligand 2 (MCP1), and lower levels of macrophage inflammatory protein 1b and IL4, indicating that mortality was associated with elevated systemic markers of inflammation in children with CSM.

### Integrative analysis reveals correlations between inflammatory markers and SCFAs and lysophospholipids associating with mortality

We investigated whether the 32 differential metabolites and 22 proteins could be mapped on known metabolome-proteome reaction models using the IMPALA (Integrated Molecular Pathway Level Analysis) database. However, no direct common pathways and interactions were found.

**Fig. 4.**
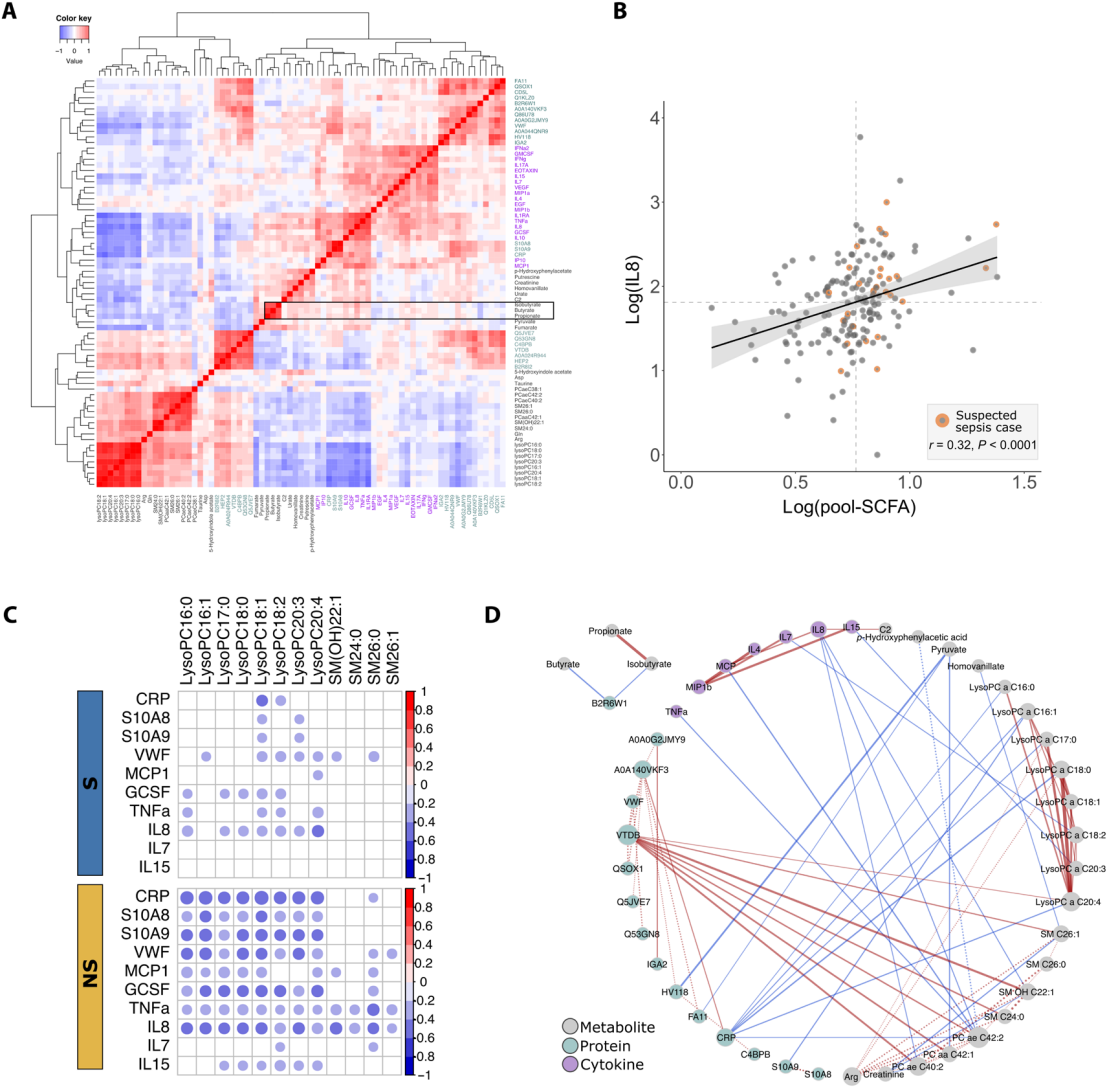
Cross-correlations of differential analytes. (**A**) Heatmap of hierarchical clustering of pairwise correlation among 32 differential metabolites (gray labels), 22 differential proteins (green labels), and 19 measured inflammatory mediators (purple labels) in all subjects. Correlations of SCFAs are highlighted in the black rectangle. (**B**) Correlations of pool SCFAs (sum of propionate, isobutyrate, and butyrate) with IL8. Dashed lines denote median levels of SCFA and IL8. (**C**) Group-specific correlations between lipids and inflammatory mediators and acute phase proteins among S compared to NS. Significant (*P*_FDR_ < 0.05) correlations are represented by circles. (**D**) Differential correlation network. Nodes are analytes, and edges connect analyte pairs with differential correlations between groups (*P*_∆r_ < 0.05). Solid edges denote increased and dashed edges denote reduced strengths of correlation in the NS, with their relative widths corresponding to the absolute difference in correlation strengths. Since no reversed correlation was detected, positive correlations are indicated by red edges and negative correlations are indicated by blue edges. Node color and size correspond to analyte classes and node degree, respectively.

We next performed cross-correlation analysis on the differential metabolites, proteins, and inflammatory mediators. Hierarchical clustering recapitulated biochemical class memberships of the analytes (Fig. 4A). For instance, lipids correlated with each other to form a cluster, while inflammatory mediators clustered together with the acute phase protein CRP and calprotectin (S100A8/A9). Several organic acids coclustered with the inflammatory mediators, including the three highly elevated SCFAs in the NS. These SCFAs appeared to be most prominently correlated with cytokine IL8, a key cytokine involved in neutrophil recruitment and degranulation. Given that SCFAs are microbial products and the role of IL8 in host response to bacterial infections, we examined the relationship between pooled SCFA (sum of propionic, isobutyric, and butyric acids) and IL8. As shown in Fig. 4B, children with higher pooled SCFA levels had higher IL8 levels (*r* = 0.3, *P* < 0.0001). We noted that 30% of the children with pooled SCFA and IL8 levels above the medians had been recorded as suspected sepsis cases by admitting clinicians, compared to only 8% suspected sepsis cases among those with either pooled SCFA or IL8 levels below the medians.

While correlations consistent in both groups may represent conserved analyte interactions, differences in group-specific correlations reflect changes in the underlying pathophysiology. Differences in within- and between-class correlations were observed between NS and S. A number of within-class correlations were altered among NS, typified by reduced protein-protein correlations and increased cytokine-cytokine correlations (fig. S5). Most between-class correlation differences were found between long-chain lipids and inflammatory mediators. Long-chain lipids uniformly showed increased negative correlations with inflammatory mediators (CRP, calprotectin, MCP.1, GCSF, TNFα, IL7, IL8, and IL15) among children in NS compared to S (Fig. 4C). Differential correlation analysis confirmed statistical significance of these observations as illustrated by the presence of edges in the differential network (Fig. 4D). For instance, the acute phase protein, CRP, which is a key network hub (high-node degree), exhibited a negative correlation with lysoPC a C20:4 among NS but an insignificant correlation among S (*r*_NS_ = −0.5, *r*_S_ = −0.2, *P*_∆r_ = 0.02). In contrast, VTDB, which is involved in immune activation processes in addition to being a vitamin D carrier, exhibited positive correlations with long-chain lipids [lysoPC C20:4, SM(OH)C22:1, SM C24:0, and PCaaC42:1] in the NS, but not in the S (*P*_∆r_ < 0.05). Hence, there was an enhanced interaction between lysophospholipids and inflammatory mediators among the NS.

### Discriminant signatures identified are substantiated by unsupervised learning on integrated clinico-omics data

**Fig. 5.**
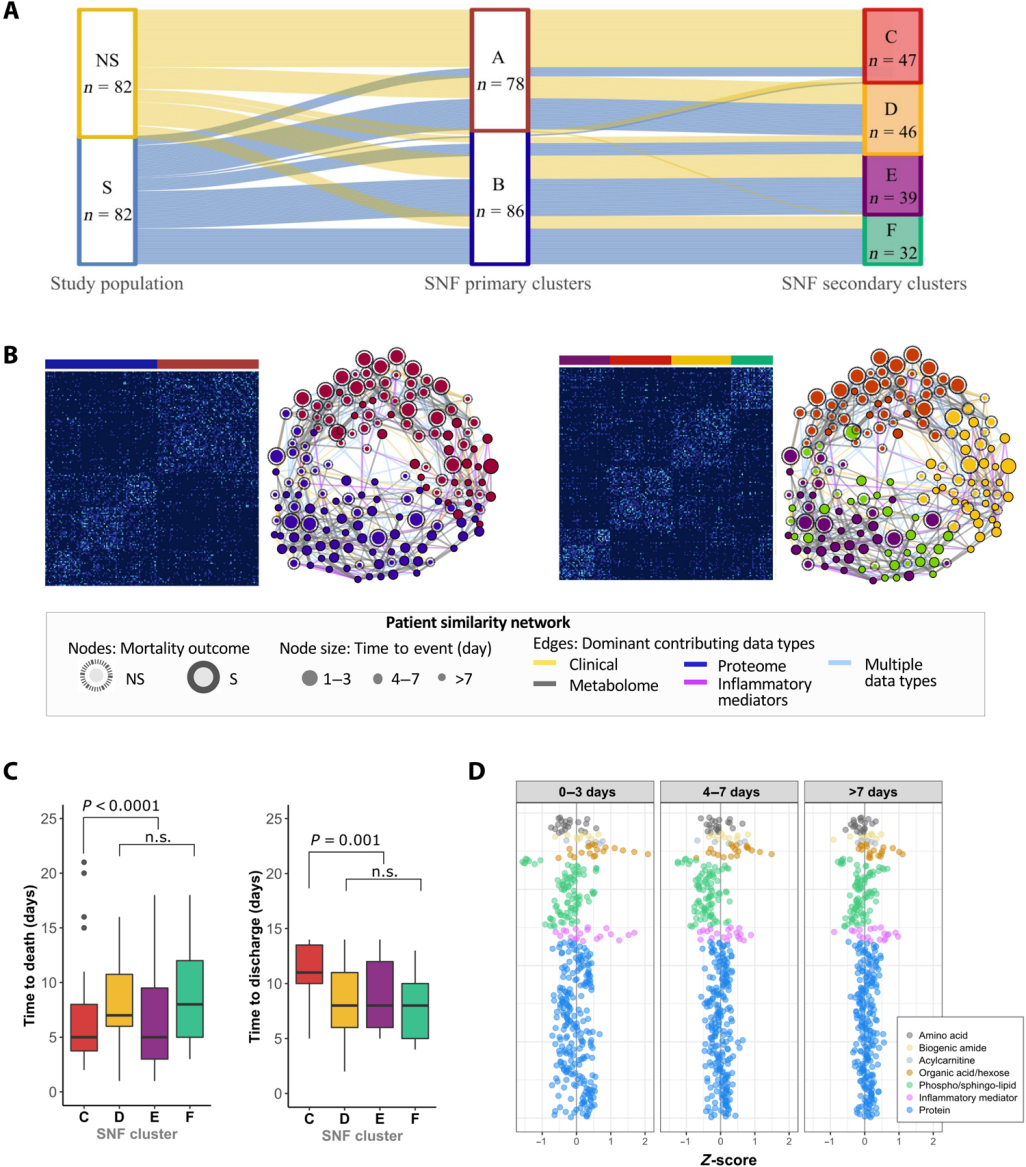
Unsupervised integrative clustering of patients with CSM by similarity network fusion (SNF). (**A**) Alluvial plot tracing clustering path of study subjects based on the combined set of clinical variables, metabolites, proteins, and cytokines quantified. (**B**) Patient similarity matrix and network based on the two and four SNF clusters and their associated network representation. In the patient-similarity network, each circle denotes an individual; edges connecting individuals are weighted by the similarity score and colored by contributing data type(s) as per legend. (**C**) Differences in time to death and time to discharge of subjects grouped into SNF clusters C, D, E, and F. Statistical test was performed by Cox proportional hazard analysis of event time. n.s., not significant. (**D**) *Z*-score scatterplots of metabolites, proteins, and inflammatory mediators of children who died during the first 3 days, during day 4 to day 7, and beyond 7 days of hospitalization. Zero on the *x* axis represents the mean of children who survived from the respective analyte. The higher the *Z*-score, the more deviated the analyte is in NS with respect to S.

Given potential interactions between clinical manifestations and biomolecular changes, as a test for robustness, we performed similarity network fusion (SNF) on the complete clinico-omics data including clinical admission information (Table 1) and biomolecular data (144 metabolites, 229 proteins, and 19 inflammatory mediators). On the basis of this unsupervised integrative analysis, study subjects could be clustered into two primary clusters (A and B) (Fig. 5A). Cluster A was dominated by NS (67%), while cluster B was dominated by S (65%). Features characterizing the death-dominated cluster (fig. S6) corroborated with discriminant features identified earlier (e.g., higher propionic acid and CRP together with lower lysoPCs). Thus, the distinct patterns between NS and S were recapitulated by the unsupervised method. The patterns were predominantly driven by differences in biomolecular signatures (concordance to fused network = 0.7) rather than clinical features (concordance to fused network = 0.1), as illustrated by edge color in patient-similarity network (Fig. 5B). This means that clinical features did not identify all individuals with underlying death-related biomolecular changes. Moreover, this analysis also revealed underlying heterogeneity in patterns linked to mortality as subjects could alternatively be grouped into four clusters (C, D, E, and F). Clusters D and E had an almost even mix of NS and S, representing subgroups of children who did not exhibit strong death-related clinico-omics signals. Children in cluster D tended to be older and edematous (60%) and had worse amino acid and lipid status than others (fig. S6). Children in cluster E tend to be younger and nonedematous (100%) and have better amino acid and lipid status than others (fig. S6). The uncovering of these two clusters suggests that specific biomolecular characteristics were associated with edema and age, although these characteristics were not linked to mortality. Cluster C was highly dominated by NS (85%), whereas cluster F was dominated by S (72%), representing subgroups of children with strong death-related clinic-omics signals than those in other clusters. Children grouped into death-predominant cluster C showed increased inflammatory mediators and decreased lysoPCs and specific amino acids, consistent with the top discriminant features identified earlier (fig. S6). This death-predominant cluster consisted of children who died earlier (IQR: 4 to 7 days) or survived but remained in the hospital longer (IQR: 10–14 days) than other clusters, as depicted by circle sizes in the patient-similarity network of Fig. 5B, which was statistically confirmed (Fig. 5C). *Z*-scores representing how much the omic profiles of NS subjects deviate from S subjects showed that children who died early had the largest differences from those survived (Fig. 5D).

## DISCUSSION

**Fig. 6.**
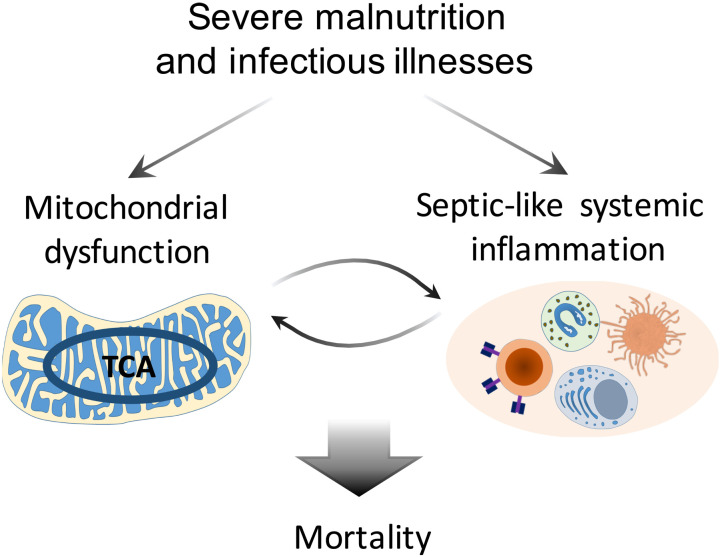
Proposed pathways underlying mortality of CSM. Mortality in children with CSM can be attributed to the interplay between mitochondrial dysfunction and systemic inflammation. Severe malnutrition leads to defective mitochondrial energy metabolism, which increases individual’s susceptibility to impaired immune response against infections leading to sepsis-like systemic inflammation. Increased systemic inflammation, in turn, amplifies bioenergetic inefficiency. The interplay between bioenergetic failure and exacerbated inflammation can cause tissue and organ damages and ultimately lead to death.

This study aimed to characterize pathways associated with inpatient mortality in children with CSM, a population with a high risk of death. We showed that among children with comparable anthropometry and HIV status upon hospital admission, those who subsequently died in hospital had increased baseline levels of TCA cycle metabolites, acetylcarnitine, and acute-phase and proinflammatory proteins and cytokines, and decreased levels of specific amino acids, lysophospholipids, and sphingolipids. Consistent results were found in sensitivity analyses assessing further adjustment for nutritional edema status and using an integrative unsupervised method. The matched case-control design allowed us to eliminate effects simply due to differences in nutritional status between survivors and nonsurvivors. These findings indicate that death is associated with altered energy metabolism related to mitochondrial dysfunction and inflammatory host responses, which are not treatment targets being emphasized by the current recommended guidelines. Overall, the present study provided first biomolecular evidence in support of the hypothesis that bioenergetic defects and overwhelming systemic inflammation underlie inpatient mortality of CSM (Fig. 6).

Metabolic flexibility of mitochondria is required for complete utilization of nutritional building blocks to maintain energy homeostasis in the face of acute illnesses and severe malnutrition (*16*, *17*). Alterations in amino acid metabolism, particularly reductions of urea cycle amino acids and BCAA, along with accumulations of incompletely oxidized energetic substrates (pyruvate, fumarate, α-ketoglutarate, succinate, and acetylcarnitine) involved in β-oxidation and the TCA cycle, were associated with CSM mortality. Although origins of these energetic substrates cannot be precisely pinpointed from blood sample profiling, their elevations point toward mitochondrial dysfunction in tissues including the liver. The hepatic TCA cycle is crucial for maintaining body glucose and energy homeostasis. Disproportionate correlations observed between hexoses and the TCA metabolites in the nonsurvivors may suggest disturbances in hepatic pathways of glucose metabolism. Metabolic disturbances related to hepatic mitochondrial dysfunction have been noted in uncomplicated severe malnutrition as well as in CSM (*4*, *10*, *11*, *18*). In an animal model of uncomplicated severe malnutrition, protein deprivation in the absence of infections can directly induce hepatic mitochondrial dysfunction leading to liver steatosis and ATP depletion (*11*). Similar to our finding in relation to mortality, Bartz *et al.* (*4*) reported that Ugandan children with CSM had lower levels of BCAA and arginine and higher levels of ketones and acylcarnitines on admission compared to after nutritional stabilization. Reduction in BCAA has been reported during prolonged starvation, and arginine is a conditionally essential amino acid that becomes essential under stress and catabolic states. Abnormal accumulations of acyl–coenzyme A (CoA), products of fatty acid β-oxidation, in mitochondria are exported to blood as acylcarnitines. Increased levels of acylcarnitines or ratios between acylcarnitines and free carnitine, therefore, imply an imbalance between β-oxidation activity and acyl-CoA oxidation (*19*). Early small liver biopsy studies among fatal cases of severe malnutrition showed that hepatic mitochondria had morphological defects that may interrupt nutrient oxidation (*10*, *18*). These similarities between our findings and previous work may reflect effects of malnutrition on mortality. However, it is not likely that these observed metabolic changes are solely attributed to nutritional causes but rather to an interplay between malnutrition, severe infection, and host inflammatory responses.

We found that inflammatory mediator proteins, calprotectin, CRP, vWF, and AGT, were higher in children who died than in those who survived. Calprotectin is predominantly expressed by neutrophils and plays a role in the inflammatory response to bacterial lipopolysaccharide by inducing neutrophil migration to inflammatory sites. CRP is a key acute phase protein produced by the liver in response to IL6, binds to damaged cell membranes and pathogens, and has a functional role in directing complement activation, opsonization, phagocytosis, and cytokine production. vWF is a biomarker of endothelial activation involved in platelet adhesion and aggregation for hemostasis and connects hemostatic and inflammatory pathways. Nonsurvivors also had increased levels of proinflammatory cytokines, IL7, TNFα, and IL8. TNFα is a multifunctional cytokine that plays a role in inflammation, immunity, and antiviral responses. IL8 is a potent chemoattractant that activates neutrophils and stimulates phagocytosis (*20*). Increased proinflammatory cytokines (IL2, IL6, and TNFα) were previously noted to be univariately associated with inpatient mortality by Bartz *et al.* (*4*).

Metabolomic data supported the association between increased systemic inflammation and mortality. Kynurenine-to-tryptophan levels were significantly elevated in nonsurvivors compared to survivors. Kynurenine is formed from tryptophan by the enzyme indolamine 2,3-dioxygenase, which is up-regulated by proinflammatory cytokines upon immune activation. Increased kynurenine-to-tryptophan ratio has been shown to correlate with CRP in malnourished children in the MAL-ED cohort (*21*). Circulating SCFAs were significantly higher in the children who died than those who survived, and a positive correlation between SCFAs and inflammatory mediators, especially IL8, was observed. SCFAs are commonly regarded as microbial-derived metabolites. Elevated circulating SCFAs may be indicative of increased translocation of bacterial products from the intestinal microbiome to the host system. Increased intestinal inflammation and barrier dysfunction have been described in both complicated and uncomplicated severe malnutrition (*22*), and in sepsis (*23*). In line with our findings, a link between increased intestinal and systemic inflammation and mortality has been reported CSM (*12*). Higher systemic levels of propionate and isobutyrate have been noted in Salmonellosis and sepsis (*24*–*26*). Weng *et al.* (*24*) showed that in patients with sepsis, serum propionate increased with sepsis severity and is an independent predictor for inpatient 28- and 90-day mortality. Nevertheless, other possibilities for systemic SCFA augmentation cannot be excluded, such as impaired utilization by colonocytes or other organs. For instance, absorbed SCFAs are metabolized first by the liver as energy sources, while hepatic energy metabolism is hampered in sepsis (*27*).

Cross-omics analysis further substantiated the close relationship between CSM mortality and sepsis. LysoPC (C16, 17, 18, and 20) and SM (C24, 26) levels were markedly lower in children who died, and decreased levels of these lipids correlated with increased levels of inflammatory mediators (CRP, TNFa, calprotectin, etc.). Intriguingly, serum lysoPC (C16, 18, 20) were previously found to be negatively correlated with gut permeability measured by lactulose-to-mannitol ratio in Malawian children with environmental enteropathy (*28*). Lysophospholipids and sphingolipids are bioactive lipids with complex roles in inflammatory response. Lysophospholipids are produced by the action of the proinflammatory phospholipase A2 on phosphatidylcholine and have important regulatory roles in immune function, such as induction of monocyte chemotaxis and macrophage activation (*29*). Degradation of long-chain sphingomyelins can yield ceramides, which are structurally similar to lipopolysaccharide from Gram-negative bacteria and have proinflammatory and tissue-damaging effects (*30*). Although it cannot be excluded that production of these lipids from phospholipids is impaired, their reductions are likely attributed to increased degradation due to increased inflammatory activities, and such reduction may adversely promote excessive proinflammatory response. Reduced circulating lysophospholipids have been consistently reported in bacterial infections including sepsis (*31*–*33*). Plasma LysoPC (C16:0, C 18:0, C 18:1, and 18:2) were found to be decreased on admission and normalized with resolution of inflammation in patients with community-acquired pneumonia (*33*). In vivo administration of lysoPC C18:0 was reported to improve survival from sepsis by enhancing neutrophil functions and bacterial clearance in a murine model (*34*). A recent meta-analysis supported the strong predictive role of reduced circulating lysophospholipids in sepsis mortality (*14*). Hence, mortality among CSM children may be associated with escalated immune activation and enhanced degradation of lysophospholipids, which can compromise host defense responses and prevent resolution of inflammation.

Systemic inflammation has been described to affect mitochondrial function. The production of reactive species during the acute-phase immune activation, such as reactive oxygen species (ROS), can directly impose damage to mitochondrial DNA and proteins in tissues and organs (*27*). Toll-like receptor 4 activation was shown to cause depletion of hepatic mitochondria DNA in a mouse model of Gram-negative bacterial sepsis (*35*). Conversely, mitochondrial dysfunction, which can be induced by malnutrition alone, can affect inflammatory responses. The TCA cycle and oxidative phosphorylation in mitochondria are main processes of generating cellular ATP and ROS. Functional mitochondrial metabolism and ROS production are necessary for T cell activation and energy-dependent bactericidal activity of macrophages (*36*). Mitochondrial dysfunction is believed to be a major determinant of mortality in sepsis (*27*). Structural derangement of mitochondria and impaired substrate utilization were seen in sepsis nonsurvivors (*37*, *38*). In a large multiomics study, Langley *et al.* (*39*) showed that plasma lactate, pyruvate, acetylcarnitines, citrate, α-ketoglutarate, and oxaloacetate were all higher in sepsis nonsurvivors than survivors upon hospital admission, reflecting incomplete substrate oxidation. A recent meta-analysis concluded that mitochondrial-related metabolites can reasonably predict sepsis mortality (*14*). Therefore, mortality from CSM and sepsis exhibits strong similarities with respect to biomolecular characteristics. It can be postulated that, compared to sepsis in well-nourished individuals, a preexisting defect in energy metabolism attributed to severe malnutrition puts children with CSM at a greater susceptibility of developing and maintaining a sepsis-like state in the face of infectious challenges, which, in turn, amplifies metabolic disturbances leading to bioenergetic failure, deterioration, and subsequent death, underscoring a malnutrition-infection synergistic relationship.

Last, other functional impairments may contribute to mortality in addition to the prominent pathways discussed above. For example, higher creatinine observed in the nonsurvivors may be indicative of a reduced renal filtration rate compared to surviving controls. Among children who died, the level of creatinine was also correlated with the other altered metabolites known to be excreted by urine, including uric acid, 5-hydroxyindoleacetate, *p*-hydroxyphenylacetate, and HVA. HVA, an end product of dopamine and norepinephrine metabolism (*40*), was highly increased in the nonsurvivors. Peripheral level of HVA is a marker for sympathetic nervous system activity. HVA levels remained significantly higher in nonsurvivors after adjusting for creatinine levels, suggesting that nonsurvivors may have a greater stress response than survivors. HVA and *p*-hydroxyphenylacetate could also be originated from the gut microbiota. Gram-negative bacteria such as *Acinetobacter* and *Pseudomonas* species can produce HVA and *p*-hydroxyphenylacetate (*41*). Elevations of circulating HVA and *p*-hydroxyphenylacetate were shown to be associated with development of septic shock and mortality in acute critical ill patients (*42*, *43*).

To our knowledge, this is the first study that comprehensively examined the multiomic profiles associated with mortality in children with CSM. In the study by Bartz *et al.* (*4*), only leptin was found to be associated with CSM mortality, but other targeted biomolecular measures such as ketones, fatty acids, creatinine, CRP, and TNF-α were not associated with mortality, which may partly be attributed to their small sample size. The present study revealed that specific and targetable pathophysiological pathways are related to mortality in this group of children. The use of a matched design to identify case-control pairs with comparable age, nutritional, and HIV status upon admission provided a priori control of these potential confounders (i.e., anthropometry, age, and HIV) for the purpose of examining proteomic and metabolomic indicators. After accounting for these factors and conducting sensitivity analyses, we showed that nonsurvivors remained robustly different from survivors. Hence, children who died exhibited substantial biomolecular differences from those who survived upon hospital admission, with those who died earlier showing the greatest differences. The hormone leptin was not identified by the proteomic panel in the current study. As mentioned above, leptin reflects fat mass and Gehrig *et al.* (*44*) previously reported that leptin correlates positively with anthropometry in malnourished children. Bartz *et al.* and Njunge *et al.* reported that low leptin levels at admission and at discharge were associated with inpatient and early post-discharge mortality, respectively, among children with CSM (*4*, *45*). With our MUAC (mid-upper arm circumference)-matched design and sensitivity analysis on WHZ, differences in adipose reserve at admission are likely to be mostly adjusted for and less likely to explain immunometabolic differences between survivors and nonsurvivors that we observed. However, a recent study reported a weak correlation between MUAC and plasma leptin, highlighting the limitations of anthropometric measures as a proxy for adipose tissue mass (*46*). Given the immunomodulating roles of leptin and imperfect existing indicators of adipose reserves, it can therefore not be excluded that lower leptin levels or reduced adipose reserves could predispose children with an increased vulnerability by affecting metabolism and immune function. To reconcile the causal relationships between leptin, substrate availability, and immune-metabolic responses in the mortality of CSM, further mechanistic investigations are warranted.

One may suspect that these biomolecular differences could be primarily attributed to clinical evidence of infection at admission, considering that children who died had a higher prevalence of diarrhea and chest indrawing. However, the integrative analysis of clinico-omic data revealed that clinical features did not explain well the observed biomolecular disturbances or mortality outcome among children in this study. Namely, clinical data only weakly contributed to the clustering of subjects in the SNF analysis, and many mortality cases did not present any clinical signs of infection. The insignificant impact of clinical features is expected, since the case-control sample by design represents a subset of children with similar clinical profiles. Also, children with CSM are thought to have blunted responses to infection and often present with multiple nonspecific symptoms varying in severity, making accurate clinical diagnosis and prognosis challenging. Overall, these findings suggest that the metabolic and immune disturbances identified represent underlying pathways associated with mortality that cannot be readily recognized on the basis of clinical evidence after accounting for nutritional status. Notably, although the current matched case-control design has strengths in addressing our primary objective of interest, it inevitably has statistical limitations when used for other purposes. Thus, assessing the precise effects of individual clinical features or comparing predictive performance between clinical and biomolecular features is beyond the scope of the study.

This study is not without limitations. First, post-discharge follow-up was not performed in the parent trial. Some of the children in the survivor group may have died shortly after discharge, as has been reported (*45*). Nevertheless, we excluded children who had prolonged hospital stay of over 14 days before being discharged to help mitigate the chance of including very sick children as controls. Second, only admission samples were analyzed, while admission samples do not necessarily reflect pathophysiological events that took place closer to the time of death. For instance, new onset of hospital-acquired Gram-negative infections could lead to sepsis and death in hours but would not be captured by the admission samples. SNF clustering revealed relationships between omic disturbances and time to death. Third, identification of the death-related biological processes was likely constrained by the limited coverage of the biomolecular panels and by the current search spaces of the public databases, although our targeted metabolomic and inflammatory mediator panels were selected to examine specific pathways hypothesized to be important in mortality of CSM. Last, this is a secondary study constrained by data and samples collected from the clinical trial. For instance, micronutrients that may provide insights into pathways were not measured because of limited sample volume. Although clinician-suspected septic cases were recorded in the trial, and these cases tend to coincide with children of sepsis-like omic profiles, these suspected cases were not confirmed using standardized criteria or routine blood cultures due to limited resources. Further external validation and targeted mechanistic studies are warranted.

This study revealed that children with CSM who died exhibited a metabolic and proteomic profile akin to sepsis. We proposed that mitochondrial-driven metabolic dysfunction previously reported in malnutrition studies likely predisposes malnourished children to develop a sepsis syndrome when faced with an infectious or proinflammatory challenge. Our findings have important clinical and treatment implications relating to antimicrobial therapy and other considerations in sepsis, including effects of feeding and pharmacological strategies to boost mitochondrial function and promote energy homeostasis, and to rebalance the inflammatory responses (*47*). Children with CSM are often only identified as severely malnourished at the time of admission, with children being directed to nutrition rehabilitation units where emphasis is placed on addressing nutritional factors, while providing empirical antibiotic treatment and supportive medical management. Our finding on a sepsis-like profile underscores that besides optimizing the use of antibiotics (dosage, duration, and timing of switching to second-line agents), many other considerations in treating sepsis should also be incorporated into the treatment of children with CSM, for instance, the potential use of immunomodulatory interventions. Overall, this study reveals a deeper biological understanding of their mortality, providing evidence that efforts to reduce mortality among sick children with severe malnutrition admitted to hospital should focus on addressing the metabolic and inflammatory disturbances, as well as infectious and immune causes of sepsis.

## MATERIALS AND METHODS

### Study design and subjects

This was a nested case-control study among children with CSM enrolled in a multicenter randomized control trial (NCT02246296). The trial investigated the effect of a lactose-free, low-carbohydrate milk formulated to limit carbohydrate malabsorption, diarrhea, and refeeding syndrome among children hospitalized for CSM in Queen Elizabeth Central Hospital, Malawi; Kilifi County Hospital; and Coast General Teaching and Referral Hospital, Mombasa, Kenya (*48*). Briefly, the trial enrolled children aged 6 months to 13 years at admission to hospital if they had CSM, defined as MUAC < 11.5 cm or weight-for-height *Z*-score < −3 if younger than 5 years old, BMI-for-age *Z*-score < −3 if older than 5 years old, or kwashiorkor edematous malnutrition at any age and had medical complications or failed an appetite test according to the WHO guidelines (*2*). Children were excluded if they had known allergy to milk products or did not provide consent. The primary outcome of the trial was the time to initial stabilization, defined as having reached the “transition” phase of treatment and switched to a standard higher caloric feed based on WHO guidelines. Of the 843 children enrolled, 8.9% died prior to stabilization and 6.2% died after the first stabilization. There was no difference in the primary outcome or mortality between the randomization arms.

This nested study used clinical variables (Table 1) and blood samples collected from study participants on admission (before randomization) and stored at −80°C. Cases (NS) were children who died during hospitalization, while controls (S) were children discharged alive within 14 days of hospitalization. S were matched with NS using propensity scores composed of age, HIV, and MUAC. Specifically, we used nearest neighbor matching as implemented in the R package “MatchIt” to identify the closest control subject for a given case subject based on the distance of their propensity scores. Diagnoses were performed on the matched sample to ensure that the distribution of the matched covariates (age, HIV, and MUAC) was similar between cases and controls (*49*). All available samples from NS (*n* = 92) were analyzed (fig. S7). For a metabolomic reference, an additional 10 nonstunted healthy community (HC) subjects were included (table S4). Baseline characteristics of subjects were summarized as medians with interquartile ranges (IQRs) or means ± the SDs for continuous variables and percentages for categorical variables.

### Metabolomic profiling

Metabolomic profiling was performed on serum collected at admission of 90 case-control pairs using two targeted platforms to measure a total of 206 metabolites. An aliquot of 25 μl was analyzed using a commercial kit (AbsoluteIDQ p180 Kit; Biocrates Life Sciences AG, Innsbruck, Austria), which quantifies 188 metabolites including 22 amino acids and 21 biogenic amines by liquid chromatography and tandem mass spectrometry (LC-MS/MS) and 40 acylcarnitines, 90 glycerophospholipids, 15 sphingolipids, and 1 hexose (sum of hexoses, representing 90 to 95% glucose) by flow injection analysis as previously described (*5*). Another 80 μl was used to measure 18 organic acids (e.g., glycolytic intermediates and TCA cycle metabolites) using the LC-MS/MS–based TMIC PRIME assay (TMIC, Edmonton, Canada). Preprocessing steps of methanol deproteination and filtration were performed in-house before samples were shipped for analysis to The Metabolomics Innovation Centre.

### Proteomics and inflammatory markers profiling

Untargeted proteomics and targeted inflammatory profiling were performed on plasma collected at admission of 87 case-control pairs. Using mass spectrometry, proteins were identified and quantified as previously described (*45*). Briefly, 10 μl of plasma was depleted of the 12 most abundant proteins using spin columns (Thermo Fisher Scientific, Rockford, USA) following the manufacturer’s instructions. Labeled peptide pool samples were generated using the Tandem Mass Tag 10-plex kit (Thermo Fisher Scientific, Illinois, USA) and desalted using ZipTips (Millipore, Darmstadt, Germany) according to the manufacturer’s instructions. Chromatographic separation of peptides was carried out on the Dionex Ultimate 3000 nanoflow liquid chromatography system on a 75 μm by 50 cm C18 reverse-phase analytical column and measured using a Q Exactive Orbitrap mass spectrometer coupled to the chromatography system via a nanoelectrospray ion source (Thermo Fisher Scientific, Illinois, USA).

Concentrations of 29 inflammatory mediators were quantified using a human cytokine magnetic bead assay (EMD Millipore, Burlington, Massachusetts, USA) on the Magpix with Xponent software (version 4.2; Luminex Corp) and acquired median fluorescent intensity data analyzed using the Milliplex analyst software (version 3.5.5.0 standard). Table S5 lists all cytokines assessed.

### Data preprocessing

For metabolomics, metabolites that had coefficient of variation <30% in quality control samples and were detected in at least 80% of samples in either group were retained for subsequent analysis. Among the retained metabolites, nondetected values were imputed by the lowest limit of detection (LOD/2). Two serum samples had insufficient volume for the analysis of organic acids, and, for these, bagged trees imputation from the “Caret” R package was used (*50*). For proteomics, identifiers, protein names, and gene names were extracted from the ProteinGroup Matrix file from Maxquant (*51*). Proteins not detected in >20% subjects were removed. Data imputation for samples with <20% missing values was performed using k-nearest neighbor method. The data were then batch-corrected using the combat software (*52*). Before analysis, all data were log_10_-transformed, scaled to unit variance by autoscaling, and mean-centered.

### Analysis of features associated with mortality

Both univariate and multivariate approaches were used to reveal features and differences in patterns of analytes associated with mortality. First, to identify individual analytes associated with mortality, univariate conditional logistic regression accounting for the matched design was performed. *P* values were adjusted for multiple testing using the Benjamini and Hochberg false discovery rate (FDR) correction.

Top significant analytes were selected on the basis of criteria as follows: FDR-corrected *P* value (*P*_FDR_) < 0.01 for metabolomics or *P* < 0.05 for proteomics. Then, an elastic net penalized logistic regression model was implemented on each omic dataset using the “glmnet” R package (*53*). Elastic net is a regularization method that simultaneously models all analytes while penalizing variable coefficients by both sum of squared coefficients and sum of absolute coefficients. This prevents overfitting due to high dimensionality and simultaneously allows for feature selection. Bootstrap resampling was used to evaluate the robustness of selected analytes. Bootstrapping was performed at 200 iterations on the elastic net model with the optimized regularization value (α = 0.75) and analytes selected by the model for more than 70% of times were considered as influential analytes. Tuning of λ was done on the basis of fivefold cross-validated misclassification error within each bootstrap sample, as previously described (*54*).

Considering that both univariate and multivariable analyses are relevant in understanding biological pathways, analytes that were statistically significant in univariate analysis or influential in multivariable analysis were considered as differential analytes. The differential analytes were then included in a partial least square discriminant analysis (PLS-DA) implemented in the “mixOmics” R package (*55*) to better visualize their interrelationships and confirm the metabolomic or proteomic signature in differentiating NS from S while also considering the matched design by using multilevel modeling. Tenfold cross-validation was used to assess the optimal number of components and the distributions of performance statistics: model fit (DQ^2^) and classification performance (AUC and misclassification rate) (*56*). The schematic of the analytical procedures is shown in fig. S8.

### Pathway analysis

To gain functional insight into pathways affected by differential omic profiles, we conducted pathway analysis. For metabolomics, we used the Pathway Analysis module on the MetaboAnalyst, which integrates pathway enrichment and topology analyses (*57*). The list of all detected metabolites in our targeted platforms was used as background reference. The conversion from common metabolite names to KEGG IDs was done by the Metabolite ID Conversion module on MetaboAnalyst. Global test was used to analyze concentrations and identify subtle changes; relative-betweenness centrality was used to establish metabolite importance. A complementary analysis of 18 biologically relevant metabolite ratios (table S1) was conducted using conditional logistic regression as described above. For proteomics, the GO-enriched biological processes of differentially expressed proteins were determined using The Database for Annotation, Visualization, and Integrated Discovery v6.8 Bioinformatics Resource (*58*).

### Integrative analysis of metabolomic and proteomic datasets

To determine whether the differential metabolite and protein features could be mapped onto known metabolome-proteome reaction models (substrate-enzyme-product), we analyzed the dataset using IMPALA [version 12 (*59*)]. IMPALA provides a pathway overrepresentation and enrichment analysis functionality with user-specified lists of genes/proteins and/or metabolites.

Network analysis of multiomics data has been shown to provide a unique view in understanding biological systems. It exploits the property that biological molecules (e.g., proteins and metabolites) tend to behave in an orchestrated manner and exhibit interconnected relationships as a network. To identify correlation patterns associated with mortality, we performed differential correlation analysis on the omics data. Pairwise Pearson’s correlation coefficients across the differential analytes (metabolite, protein, and cytokines) were separately computed within the NS and S group. Analyte pairs were considered as correlated if their correlation coefficient was significant at *P*_FDR_ < 0.05 in either the NS or S group. Differences in correlation strengths were subsequently compared between NS and S after the Fisher’s z-transformation at *P* < 0.05 using the “corTest” R package. The differential correlation network was visualized using Cytoscape v3.7.2 (*60*). Network properties were described using the “NetworkAnalyzer” tool integrated in Cytoscape. The computation of correlation matrices and network properties are detailed in Supplementary Materials and Methods.

### Sensitivity analyses

To examine the robustness of our findings, the following sensitivity analyses were conducted: (i) Univariate regression models were additionally adjusted for admission edema status or WHZ, considering that these features may confound the relationship between metabolites and mortality; (ii) multivariable analysis was performed on edema-adjusted or WHZ-adjusted residuals; (iii) influential observations were inspected based on PCA, hierarchical clustering with single linkage, and univariate distributions; discriminant analyses were repeated with them removed. Moreover, the main analysis used supervised learning methods. Thus, we performed the SNF, an unsupervised integrative multiomics analysis, using the complete metabolomic, proteomic, and clinical data. The goal of the SNF analysis was to (i) evaluate whether the differences between NS and S were robust enough to be revealed in an unsupervised framework without prior feature selection and (ii) reveal potential unrecognized patient subgroups as our population is known to be heterogeneous. The SNF analysis is described in detail in Supplementary Materials and Methods.

## Supplementary Materials

### This PDF file includes:

Supplementary Materials and Methods Figs. S1 to S8 Tables S1 to S5 References

## Notes

View/request a protocol for this paper from *Bio-protocol*.
