# Supplementary material for: Systemic inflammation and metabolic disturbances underlie inpatient mortality among ill children with severe malnutrition
Source: Sci Adv. 2022 Feb 16;8(7):eabj6779. doi: 10.1126/sciadv.abj6779 (PMC8849276; doi:10.1126/sciadv.abj6779)
Supplement: Supplementary file 1 — Supplementary Materials and Methods Figs. S1 to S8 Tables S1 to S5 References [file sciadv.abj6779_sm.pdf]

Science Advances
AAAS

Supplementary Materials for

Systemic inflammation and metabolic disturbances underlie inpatient mortality among ill children with severe malnutrition

Bijun Wen, James M. Njunge, Celine Bourdon, Gerard Bryan Gonzales, Bonface M. Gichuki, Dorothy Lee, David S. Wishart, Moses Ngari, Emmanuel Chimwezi, Johnstone Thitiri, Laura Mwalekwa, Wieger Voskuijl, James A. Berkley, Robert HJ Bandsma*

*Corresponding author. Email: robert.bandsma@sickkids.ca

Published 16 February 2022, *Sci. Adv.* **8**, eabj6779 (2022)
DOI: 10.1126/sciadv.abj6779

This PDF file includes:

Supplementary Materials and Methods
Figs. S1 to S8
Tables S1 to S5
References

Supplementary Materials and Methods

Plasma proteomics

Individual plasma samples (10µl) were depleted the top 12 abundant plasma proteins using spin columns (Thermo scientific, Illinois, USA) following manufacturer's instructions. The volume of the flow through was reduced to ~50µl using the Speedvac concentrator (Thermo Scientific, Illinois, USA) and protein concentration determined using Bradford assay (Bio-Rad, California, USA). Proteins samples (20µg) were adjusted with 100mM Triethylammoniumbicarbonate (TEAB, Sigma-Aldrich) to 100µl. The protein solution was then reduced with 40mM Dithiothreitol (Sigma-Aldrich, Missouri, USA) at 65°C for 1 hour and subsequently alkylated in the dark at room temperature for 1 hour with 80mM iodoacetamide (Sigma-Aldrich, Missouri, USA). Proteins were precipitated for 1 hour at -20 °C with four volumes of pre-chilled (-20 °C) acetone (Sigma-Aldrich, Missouri, USA). The samples were then centrifuged for 10 min at 15,000 x *g* at room temperature and the supernatant discarded. The acetone-precipitated protein pellet was air-dried for 5 minutes at room temperature and resuspended in 100µl of 100mM TEAB. Trypsin (Thermo Scientific, Illinois, USA) was added to the protein samples at a trypsin-protein sample ratio of 1:20 and protein digestion was allowed to proceed overnight (16 hours) at 37°C with shaking. A pooled sample was prepared by combining 1 µl aliquot from each sample. Peptide samples were individually labelled using the Tandem Mass Tag (TMT) 10-plex kit (Thermo scientific, Illinois, USA) according to manufacturer's instructions. Two isobaric tags were exclusively used to individually label two pooled control samples. The labelled peptides for the 8 individual tags in the 10-plex were subsequently combined to generate individual pools upon which the common pool was equally distributed. The volume of each pool was reduced to ~150µl using the Speedvac concentrator. The labelled peptide pools were desalted using P10 C18 pipette ZipTips (Millipore, Darmstardt, Germany) according to manufacturer's instructions. Eluted peptides were dried in a Speedvac concentrator and re-suspended in 15µl loading solvent (97.05% H_2O, 2% acetonitrile, 0.05% formic acid). Peptides were quantified using Qubit Protein Assay Kit (Thermo Scientific, Illinois, USA) as per the manufacturer's instructions prior injection and 4µg were used for LC-MS/MS analysis.
Peptides (4µg) were loaded onto a Dionex Ultimate 3000 nano-flow ultra-high-pressure liquid chromatography system (Thermo Scientific, Illinois, USA) with a 75µm x 2cm C18 trap column (Thermo Scientific, Illinois, USA). Chromatographic separation of peptides was carried out on a on a 75 µm × 50 cm C18 reverse-phase analytical column (Thermo Scientific, Illinois, USA) at 40 °C. Elution was carried out with mobile phase B (80% acetonitrile with 0.1% formic acid) gradient (2 to 35% B) over 360 min at a flow rate of 0.3 µl/min. Each LC run was finished by washout with 98% B for 5 min and re-equilibration in 4% B for 12 min to avoid sample carryover. Peptides were measured using a Q Exactive Orbitrap mass spectrometer (Thermo Scientific, Illinois, USA) coupled to the chromatography system via a nano-electrospray ion source. The MS^1 settings were: Resolution, 70000; Automatic gain control (AGC) target, 3e6; maximum injection time, 120ms; scan range, 380-2000m/z; while the MS^2 settings were: Resolution, 35000; AGC target, 5e4; maximum injection time, 120ms; isolation window, 2.0 m/z. The top 15 most intense ions were selected for MS^2 and fragmented with higher-energy collision fragmentation using normalized collision energy of 28V and these ions were subsequently excluded for the next 30s.

Mass spectrometry protein identification and quantitation

Mass spectrometer raw files were analysed by MaxQuant software version 1.6.5.0 (*51*) by searching against the human Uniprot FASTA database (Downloaded December 2018) using the Andromeda search engine (*61*). Cysteine carbamidomethylation and TMT-10plex labelled N-terminus and lysine were set as a fixed modification and N-terminal acetylation and methionine oxidations as variable modifications. The false discovery rate (*62*) was set to 0.01 for both proteins and peptides with a minimum length of seven amino acids and was determined by searching a reverse database. Enzyme specificity was set as C-terminal to arginine and lysine with trypsin as the protease. A maximum of two missed cleavages were allowed in the database search. Peptide identification was performed with an allowed initial precursor mass deviation of up to 7 parts per million (ppm) and an allowed fragment mass deviation of up to 20 ppm. A minimum peptide length of 7 amino acids and a maximum peptide mass of 4600 Da was allowed for the searches. Other parameters were used as default settings for Orbitrap-type data. A minimum of 2 unique peptides for a protein was considered a positive identification. The 10-plex corrected reporter ion intensity matrix was extracted from the MaxQuant proteingroups output file and batch corrected using the pooled sample channels. Protein groups are clusters of proteins or protein isoforms with high sequence similarity cannot be unambiguously identified by unique peptides (but have only shared peptides) are grouped in one protein group and quantified together. Potential contaminants, protein hits from decoy database, and proteins initially depleted but subsequently detected were excluded before exporting the protein group matrix file into the analytical and manipulation environment.

Cross-correlation and differential network analysis

Pairwise Pearson's correlation coefficients were calculated separately within the NS and the S on the log-transformed data. For the integrative analysis differential analytes (32 metabolites, 22 proteins and 19 cytokines), a matrix of 3422 possible pairwise correlation coefficients was computed for each group. Similarly, for the analysis of correlation patterns focusing on metabolic pathways, matrices of 2862 possible pairwise correlations (54 metabolites) were computed. Specifically, group-specific correlation coefficients between analyte A and B for group M was defined by the following equation.

$$r_M(A,B) = \frac{\sum_{k=1}^{nM}(A_k - \bar{A})(B_k - \bar{B})}{\sqrt{\sum_{k=1}^{nM}(A_k - \bar{A})^2}\sqrt{\sum_{k=1}^{nM}(B_k - \bar{B})^2}}$$

in which $\bar{A}$, $\bar{B}$ are the mean concentrations of analyte A and B, respectively, in group M; k is the sample index and n_M is the number of samples in group M. Correlation p-values were computed using the "Hmisc" R package (http://CRAN.R-project.org/package=Hmisc). Group-specific correlation was deemed significant at FDR corrected p<0.05. Analyte pairs that were not significantly correlated in both of the study groups were removed from subsequently differential analysis. The correlation coefficients of the remaining pairs were normalized by Fisher's z transformation using the "corTest" R package (*63-65*), where the transformation was conducted as below.

$$Z_M(A,B) = \frac{1}{2}\log\frac{1+r_M}{1-r_M}$$

Statistical significance of the difference in the Z-transformed correlations between conditions M and N (i.e. NS and S) was tested by the following equation.

$$Z_{(M\ vs.N)}(A,B) = \frac{|Z_M - Z_N|}{\sqrt{\frac{1}{n_M - 3} + \frac{1}{n_N - 3}}}$$

The Z value approximate to Gaussian distribution under the null hypothesis of equal correlations.
Significant difference in correlations was tested at p<0.05. The resulting differential pairs were used to construct the differential network, which can be viewed as a net network after subtracting the case-specific network by the control-specific network.

The software Cytoscape v3.7.2 (*60*) was used to visualize the final differential network. To describe the nature of the correlation differences between groups, we defined the following relationships when plotting the edges of the differential network.

$$r(A,B) = \begin{cases} r(A,B) & if\ p_{FDR.} < 0.05 \\ 0 & if\ p_{FDR.} \geq 0.05 \end{cases}$$

$$r_M(A,B)\ vs\ r_N(A,B) = \begin{cases} correlation\ changed\ direction, & if\ r_M * r_N < 0 \\ correlation\ increased\ in\ M, & if\ r_M * r_N \geq 0, and\ |r_M| > |r_N| \\ correlation\ decreased\ in\ M, & if\ r_M * r_N \geq 0, and\ |r_M| < |r_N| \end{cases}$$

For example, if analytes A and B are significantly correlated in the same direction ($r_M * r_N > 0$) in both the NS and S ($r_M * r_N \neq 0$), and the correlation value in the NS is greater than in the S ($|r_M| > |r_N|$), then the correlation strength between A and B is considered to be increased in the NS when compared to the S. This attribute was illustrated in the network by edge line types, in which solid lines denote increased correlation strengths in the NS, dashed lines denote decreased correlation strengths in the NS, and wavy lines denote reversed correlation directions if any. To identify key analytes with important roles in the overall interrelated structure of the differential network, graph theory-based network properties were computed by the "NetworkAnalyzer" tool integrated in Cytoscape (*66*). In particular, the node degree describes the number of edges the node has. Analytes with high node degrees are highly connected, representing "hubs" of the network.

Unsupervised integrative analysis of metabolomic, proteomic, cytokine and clinical profiles

To investigate whether subjects form clusters that correspond to their survival outcomes based on the combined profile of the metabolomic, proteomic, cytokine and clinical data in an unsupervised framework, the similarity network fusion (SNF) was used (*67*). SNF has been shown to be advantageous in the integration of large-scaled datasets whether they are numerical or categorical (*68*). Given its ability to handle large-scaled datasets, the entire set of the detected analytes

were included to the analysis (i.e. no feature selection was conducted). SNF analysis also allowed us to explore contribution of different data types, including categorical clinical data, in the formation of the patient similarity network. In order to calculate patient similarity scores, SNF requires subjects to have complete data in all data types being integrated. As a result, 82 case-control pairs had complete clinical and omics data and were included to the analysis. Seven separate patient similarity matrices were first generated for each of the data types (amino acids and amides, acylcarnitines and lipids, organic acids, derived metabolite ratios, proteins, cytokines, and clinical characteristics), which were then aggregated into one fused patient similarity network using parameters K=20, alpha=0.5 and t=20. Spectral clustering was then performed on the fused matrix according to the SNFtool R package (*67*). The number of clusters were determined based on eigen-gaps and rotation cost. The final similarity network was visualized with the edge-weighted spring embedded layout in Cytoscape (*60*). Features characterizing the resulting SNF clusters were identified using logistic regressions and the normalized mutual information (NMI) score, which ranks features based on their contributions to the fused network. Clustering concordance that indicates contribution of individual data type to the final fused network was computed using the 'concordanceNetworkNMI' function of the SNFtool R package (*67*). Concordance scores are between 0 and 1, with 1 indicating perfect agreement of cluster assignments between a data type and the fused network and 0 being no agreement.

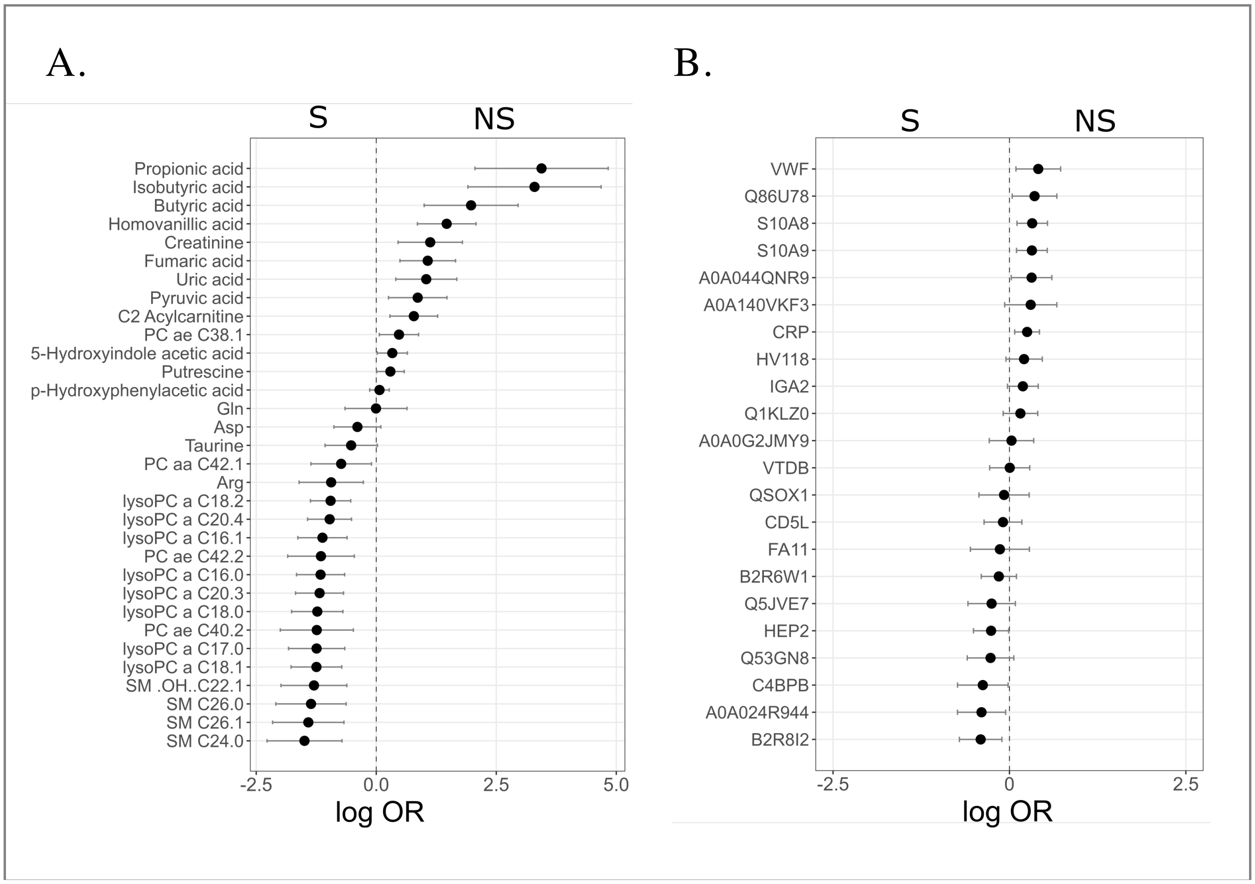

Fig. S1. Log odds ratios with 95% CI of differential analytes. (A) 32 differential metabolites. (B) 22 differential proteins.

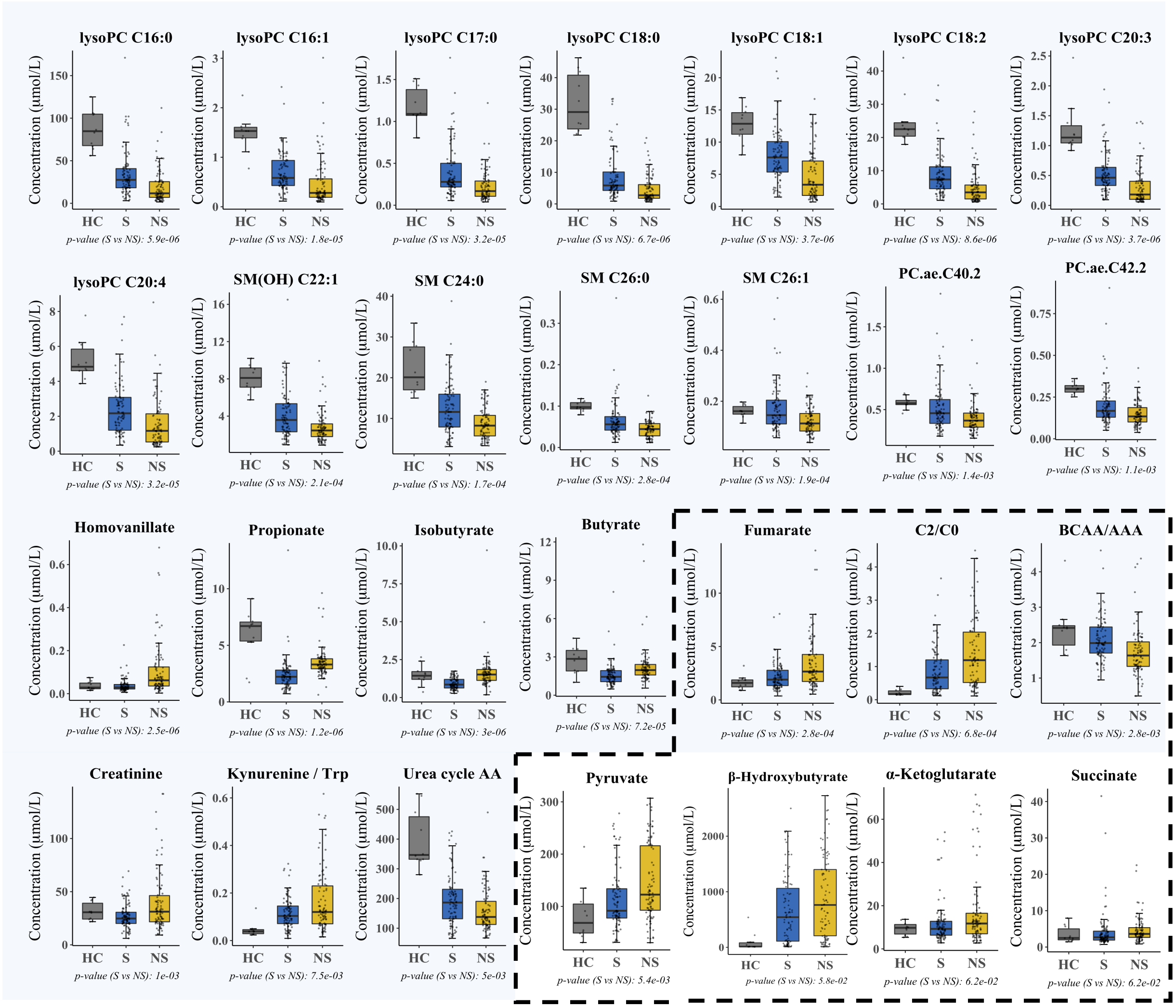

Fig. S2. Boxplots of top differential metabolites, metabolite ratios and TCA-related metabolites between S, NS and HC. Shaded area includes the top contributing metabolites identified by the PLS-DA analysis (Fig. 1D), and the significantly differential metabolite ratios between NS and S. Dashed boarder indicate metabolites related to the TCA cycle. P-values when comparing S and NS are shown beneath each metabolite. For clinical relevance of metabolic alterations between S and NS, concentrations from 10 healthy subject from the community are also plotted for reference.

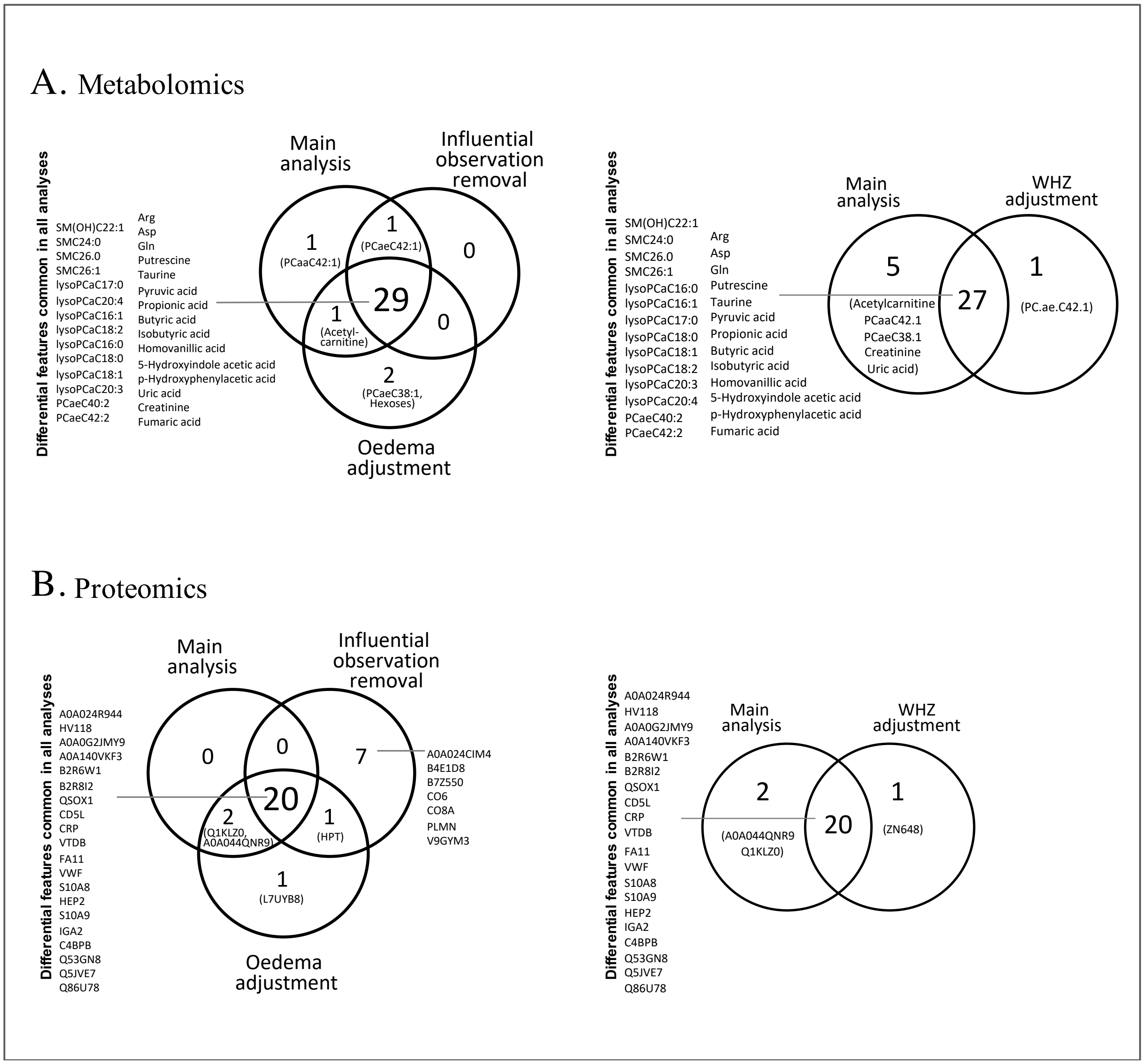

Fig. S3. Venn diagram of differential analytes identified by main analysis and sensitivity analyses. Three sensitivity analysis were performed: one with influential observation removed, one with data adjusted for the presence of nutritional oedema, and the other with data adjusted for WHZ. Differential analytes were determined by the same procedure as the main analysis, and results were compared. (A) Differential metabolites. For sensitivity analysis that removed influential observations, no subject was consistently detected as influential by PCA and hierarchical clustering; 8 values were removed based on their univariate distributions and were then imputed. These 8 extreme values (>7 standard deviations from mean) found in methionine, PCaaC36:0, PCaaC38:0, PCaaC40:2, PCaeC38:1, PCaeC38:2, SMC26:0, and propionic acid, respectively. (B) Differential proteins. For sensitivity analysis that removed influential observations 2 subjects were removed as they were consistently detected as influential by PCA and hierarchical clustering, resulting in 85 pairs of case-control data for analysis; no extreme values were detected by univariate distributions. WHZ: weight for height z score.

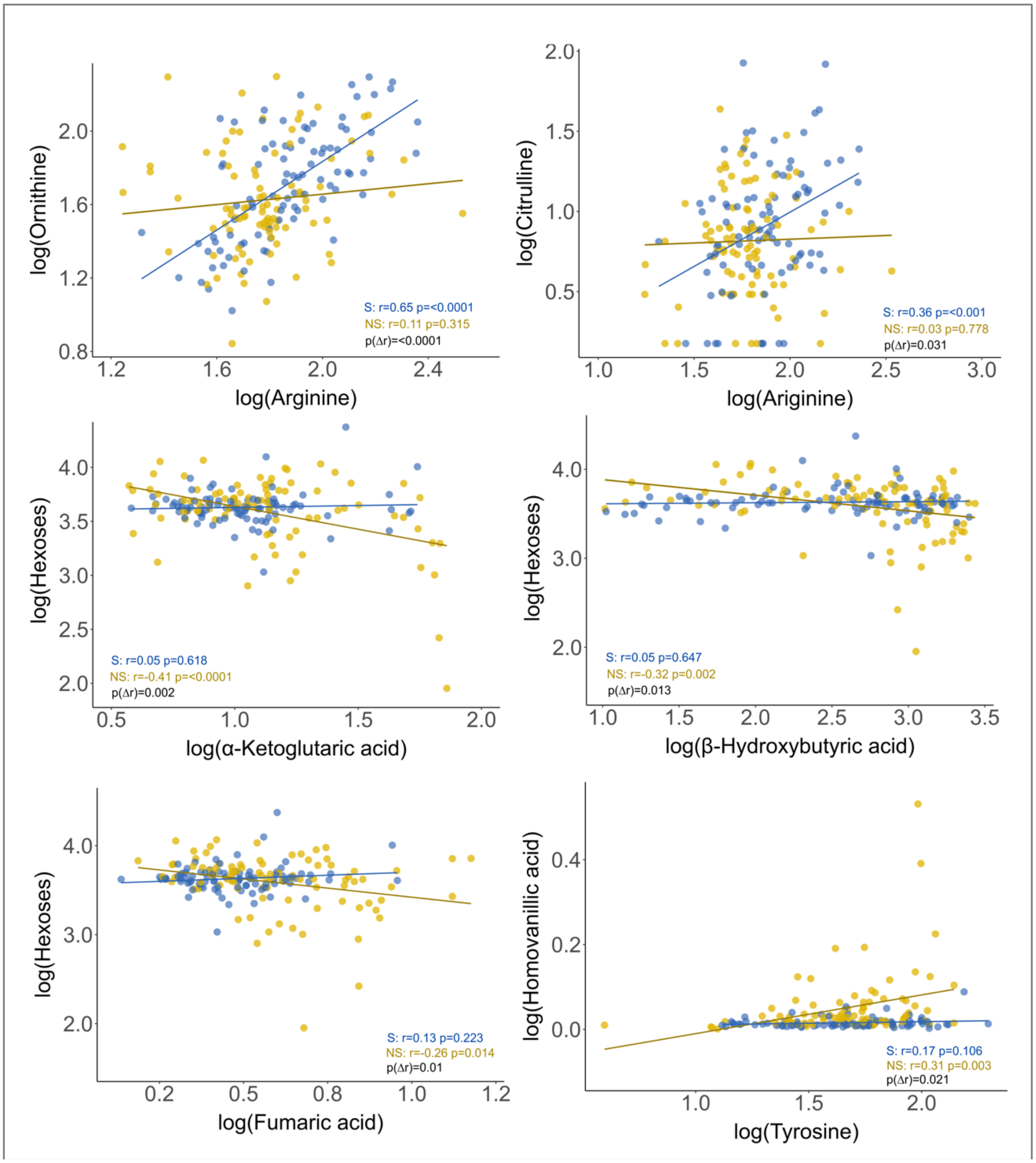

Fig. S4. Selected differential metabolite-metabolite correlations between NS and S. As a sensitivity analysis, non-parametric Spearman's rank correlation tests were also performed to confirm the Pearson correlations showed.

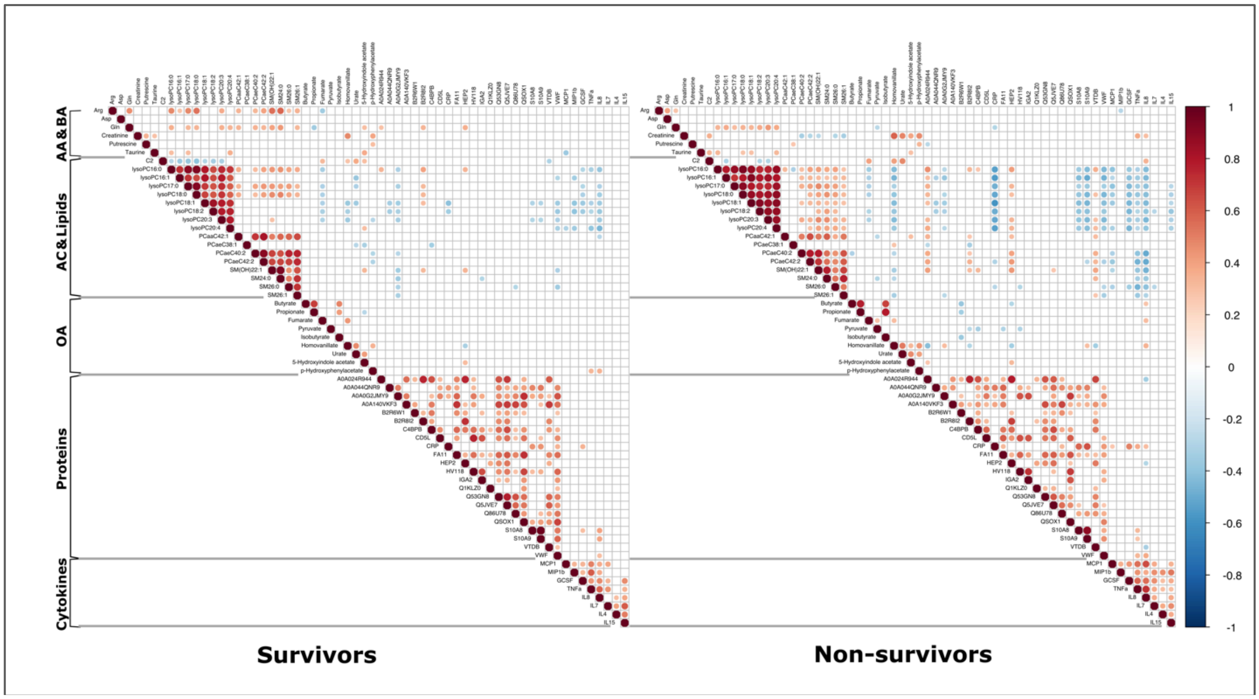

Fig. S5. Cross-correlations of differential analytes. Group-specific cross-correlation analysis of the differential metabolites, proteins and inflammatory mediators among NS and S. Significant (P_{FDR}<0.05) positive correlations are depicted in red and negative correlations are depicted in blue, with color intensity and circle size correspond to correlation strength. AA: amino acid, BA: biogenic amide, AC: acylcarnitine, OA: organic acid.

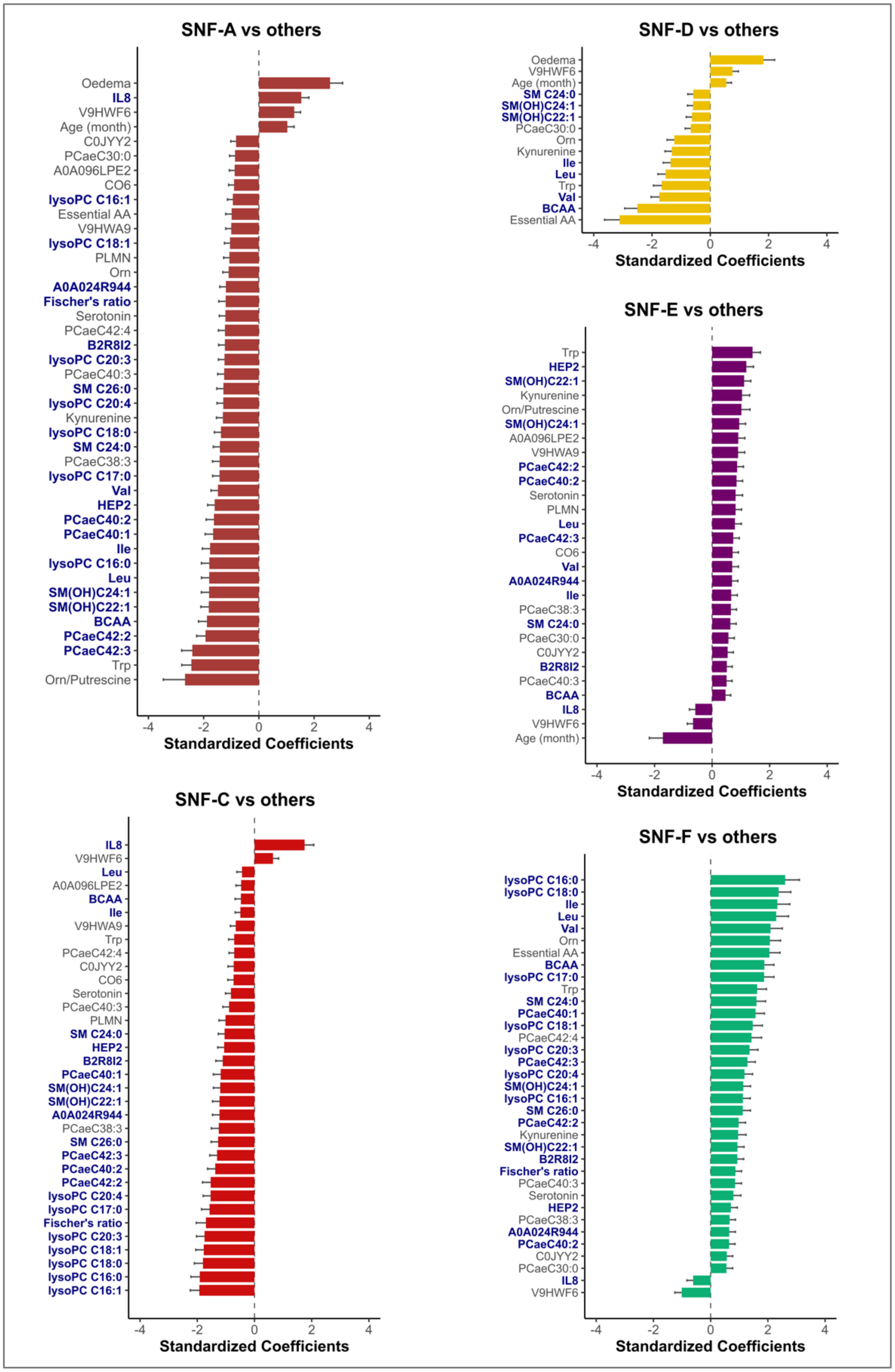

Fig. S6. Features characterizing SNF patient clusters. Standardized coefficients of top features characterizing each of the SNF clusters ($P_{FDR}<0.05$ and NMI score>90 percentile); among these top features, differential features identified by the main analysis are bolded and highlighted in blue.

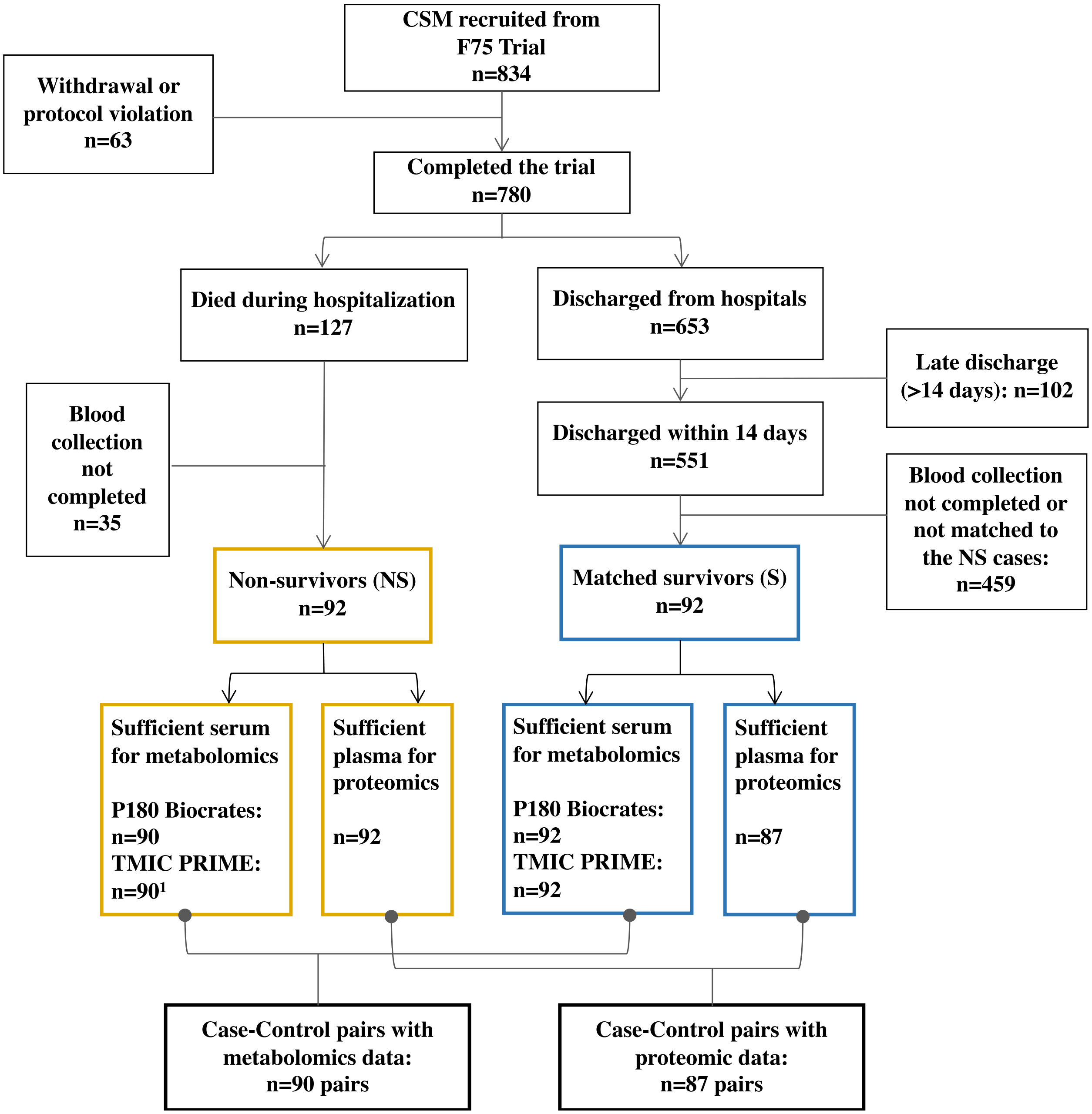

Fig. S7. Schematic of trial and case-control sample analysis.

[1] Data for 2 samples out of the 90 were imputed due to insufficient volume for TMIC PRIME, as detailed in methods. CSM: children with complicated severe malnutrition.

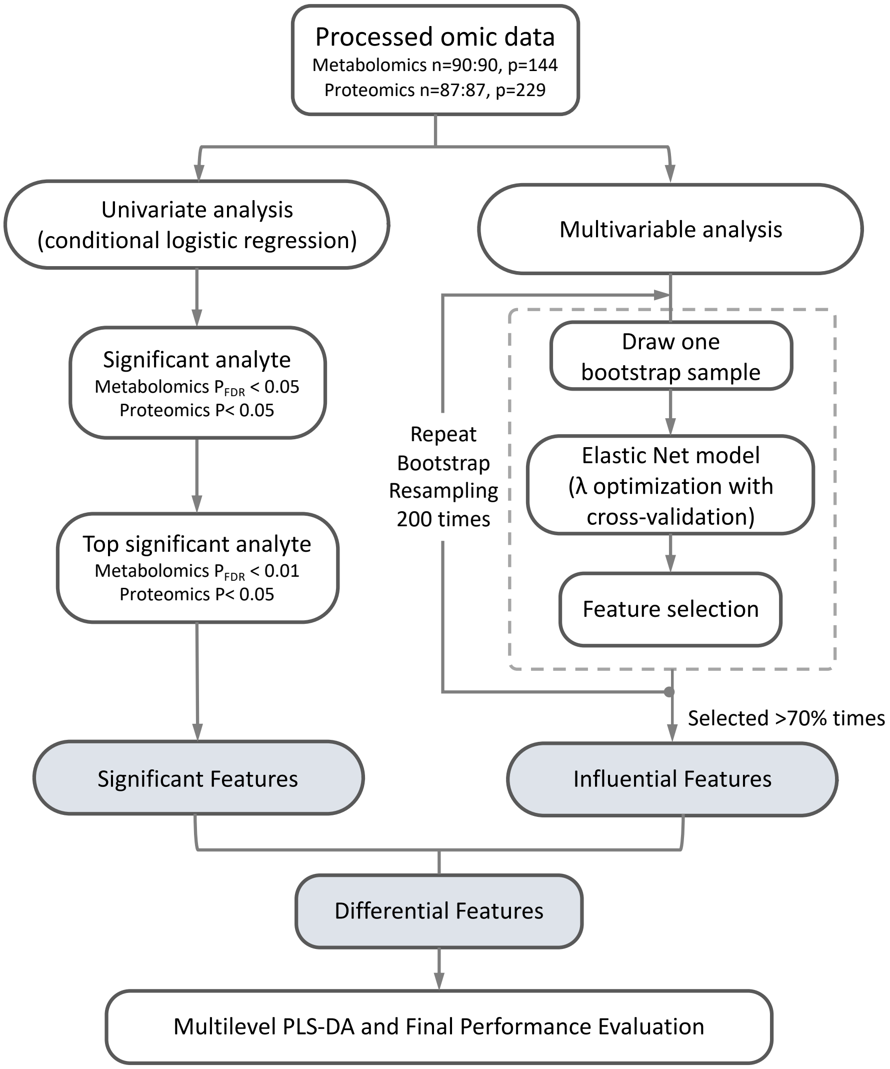

Fig. S8. Flow chart of analytical procedures.

Table S1. Metabolite detected, and ratios computed.

Metabolite and Calculated Ratios	Common name	HMDB ID	Mapped KEGG ID
Ala	L-Alanine	HMDB0000161	C00041
Arg	L-Arginine	HMDB0000517	C00062
Asn	L-Asparagine	HMDB0000168	C00152
Asp	L-Aspartic acid	HMDB0000191	C00049
Cit	Citrulline	HMDB0000904	C00327
Gln	L-Glutamine	HMDB0000641	C00064
Glu	L-Glutamic acid	HMDB0000148	C00025
Gly	Glycine	HMDB0000123	C00037
His	Histamine	HMDB0000870	C00388
Ile	L-Isoleucine	HMDB0000172	C00407
Leu	L-Leucine	HMDB0000687	C00123
Lys	L-Lysine	HMDB0000182	C00047
Met	L-Methionine	HMDB0000696	C00073
Orn	Ornithine	HMDB0000214	C00077
Phe	L-Phenylalanine	HMDB0000159	C00079
Pro	L-Proline	HMDB0000162	C00148
Ser	L-Serine	HMDB0000187	C00065
Thr	L-Threonine	HMDB0000167	C00188
Trp	L-Tryptophan	HMDB0000929	C00078
Tyr	L-Tyrosine	HMDB0000158	C00082
Val	L-Valine	HMDB0000883	C00183
alpha.AAA	Aminoadipic acid	HMDB0000510	C00956
Creatinine	Creatinine	HMDB0000562	C00791
Kynurenine	L-Kynurenine	HMDB0000684	C00328
Putrescine	Putrescine	HMDB0001414	C02896
Serotonin	Serotonin	HMDB0000259	C00780
Spermidine	Spermidine	HMDB0001257	C00315
Spermine	Spermine	HMDB0001256	C00750
t4.OH.Pro	4-Hydroxyproline	HMDB0000725	C01157
Taurine	Taurine	HMDB0000251	C00245
total.DMA	Asymmetric dimethylarginine	HMDB0001539	C03626
total.DMA	Symmetric dimethylarginine	HMDB0003334	
C0	L-Carnitine	HMDB0000062	C00318
C2	L-Acetylcarnitine	HMDB0000201	C02571
C3	Propionylcarnitine	HMDB0000824	C03017
C3.DC..C4.OH.	Hydroxybutyrylcarnitine	HMDB0013127	

C4	Butyrylcarnitine	HMDB0002013	C02862
lysoPC.a.C16.0	LysoPC(16:0)	HMDB0010382	C04230
lysoPC.a.C16.1	LysoPC(16:1(9Z))	HMDB0010383	C04230
lysoPC.a.C17.0	LysoPC(17:0)	HMDB0012108	C04230
lysoPC.a.C18.0	LysoPC(18:0)	HMDB0010384	C04230
lysoPC.a.C18.1	LysoPC(18:1(9Z))	HMDB0002815	C04230
lysoPC.a.C18.2	LysoPC(18:2(9Z,12Z))	HMDB0010386	C04230
lysoPC.a.C20.3	LysoPC(20:3(5Z,8Z,11Z))	HMDB0010393	C04230
lysoPC.a.C20.4	LysoPC(20:4(5Z,8Z,11Z,14Z))	HMDB0010395	C04230
lysoPC.a.C24.0	LysoPC(24:0)	HMDB0010405	C04230
lysoPC.a.C26.0	lysoPC(26:0)	HMDB0029205	
lysoPC.a.C26.1	lysoPC(26:1(5Z))	HMDB0029220	
lysoPC.a.C28.0	lysoPC(28:0)	HMDB0029206	
lysoPC.a.C28.1	lysoPC(28:1(5Z))	HMDB0029221	
PC.aa.C24.0			
PC.aa.C28.1			
PC.aa.C30.0	PC(P-16:0/14:0)	HMDB0011203	
PC.aa.C30.2			
PC.aa.C32.0	PC(16:0/16:0)	HMDB0000564	C00157
PC.aa.C32.1	PC(14:0/P-18:1(11Z))	HMDB0007897	C00157
PC.aa.C32.2	PC(16:1(9Z)/16:1(9Z))	HMDB0008002	C00157
PC.aa.C32.3			
PC.aa.C34.1	PC(14:0/20:1(11Z))	HMDB0007879	C00157
PC.aa.C34.2	PC(14:0/20:2(11Z,14Z))	HMDB0007880	C00157
PC.aa.C34.3	PC(14:0/20:3(8Z,11Z,14Z))	HMDB0007882	C00157
PC.aa.C34.4	PC(14:0/20:4(8Z,11Z,14Z,17Z))	HMDB0007884	C00157
PC.aa.C36.0	PC(14:0/22:0)	HMDB0007886	C00157
PC.aa.C36.1	PC(14:0/22:1(13Z))	HMDB0007887	C00157
PC.aa.C36.2	PC(18:1(9Z)/18:1(9Z))	HMDB0000593	C00157
PC.aa.C36.3	PC(16:0/20:3(8Z,11Z,14Z))	HMDB0007981	C00157
PC.aa.C36.4	PC(16:0/20:4(5Z,8Z,11Z,14Z))	HMDB0007982	C00157
PC.aa.C36.5	PC(16:0/20:5(5Z,8Z,11Z,14Z,17Z))	HMDB0007984	C00157
PC.aa.C36.6	PC(22:5(4Z,7Z,10Z,13Z,16Z)/14:1(9Z))	HMDB0008657	C00157
PC.aa.C38.0	PC(16:0/22:0)	HMDB0007985	C00157
PC.aa.C38.1	PC(20:0/18:1(9Z))	HMDB0008269	C00157
PC.aa.C38.3	PC(20:0/18:3(6Z,9Z,12Z))	HMDB0008271	C00157
PC.aa.C38.4	PC(18:2(9Z,12Z)/20:2(11Z,14Z))	HMDB0008145	C00157
PC.aa.C38.5	PC(20:1(11Z)/18:4(6Z,9Z,12Z,15Z))	HMDB0008306	C00157
PC.aa.C38.6	PC(18:2(9Z,12Z)/20:4(5Z,8Z,11Z,14Z))	HMDB0008147	C00157

PC.aa.C40.2	PC(20:1(11Z)/20:1(11Z))	HMDB0008308	C00157
PC.aa.C40.3	PC(18:1(9Z)/22:2(13Z,16Z))	HMDB0008119	C00157
PC.aa.C40.4	PC(22:0/18:4(6Z,9Z,12Z,15Z))	HMDB0008536	C00157
PC.aa.C40.5			
PC.aa.C40.6	PC(18:1(9Z)/22:5(4Z,7Z,10Z,13Z,16Z))	HMDB0008121	C00157
PC.aa.C42.0	PC(22:0/20:0)	HMDB0008537	C00157
PC.aa.C42.1	PC(18:1(11Z)/24:0)	HMDB0008091	C00157
PC.aa.C42.2	PC(18:1(11Z)/24:1(15Z))	HMDB0008092	C00157
PC.aa.C42.4	PC(18:3(6Z,9Z,12Z)/24:1(15Z))	HMDB0008191	C00157
PC.aa.C42.5	PC(20:3(8Z,11Z,14Z)/22:2(13Z,16Z))	HMDB0008415	C00157
PC.ae.C30.0	PC(o-14:0/16:0)	HMDB0013341	
PC.ae.C30.1	PC(o-14:0/16:1(9Z))	HMDB0013402	
PC.ae.C30.2	PC(o-16:1(9Z)/14:1(9Z))	HMDB0013410	
PC.ae.C32.1	PC(o-16:0/16:1(9Z))	HMDB0013404	
PC.ae.C32.2	PC(o-16:1(9Z)/16:1(9Z))	HMDB0013411	
PC.ae.C34.0	PC(o-16:0/18:0)	HMDB0013405	
PC.ae.C34.1	PC(o-16:1(9Z)/18:0)	HMDB0013412	
PC.ae.C34.2	PC(O-16:0/18:2(9Z,12Z))	HMDB0011151	
PC.ae.C34.3	PC(o-16:1(9Z)/18:2(9Z,12Z))	HMDB0013413	
PC.ae.C36.0	PC(o-16:0/20:0)	HMDB0013406	
PC.ae.C36.1	PC(o-16:1(9Z)/20:0)	HMDB0013414	
PC.ae.C36.2	PC(o-18:0/18:2(9Z,12Z))	HMDB0013418	
PC.ae.C36.3	PC(o-18:1(11Z)/18:2(9Z,12Z))	HMDB0013425	
PC.ae.C36.4	PC(o-16:0/20:4(8Z,11Z,14Z,17Z))	HMDB0013407	
PC.ae.C36.5	PC(o-16:1(9Z)/20:4(8Z,11Z,14Z,17Z))	HMDB0013415	
PC.ae.C38.0	PC(o-16:0/22:0)	HMDB0013408	
PC.ae.C38.1	PC(o-16:1(9Z)/22:0)	HMDB0013416	
PC.ae.C38.2	PC(o-18:1(9Z)/20:1(11Z))	HMDB0013431	
PC.ae.C38.3	PC(o-20:0/18:3(6Z,9Z,12Z))	HMDB0013439	
PC.ae.C38.4	PC(o-18:0/20:4(8Z,11Z,14Z,17Z))	HMDB0013420	
PC.ae.C38.5	PC(o-18:1(9Z)/20:4(8Z,11Z,14Z,17Z))	HMDB0013432	
PC.ae.C38.6	PC(o-16:0/22:6(4Z,7Z,10Z,13Z,16Z,19Z))	HMDB0013409	
PC.ae.C40.1	PC(o-18:1(9Z)/22:0)	HMDB0013433	
PC.ae.C40.2	PC(o-18:2(9Z,12Z)/22:0)	HMDB0013437	
PC.ae.C40.3	PC(o-22:0/18:3(6Z,9Z,12Z))	HMDB0013445	
PC.ae.C40.4	PC(o-20:0/20:4(8Z,11Z,14Z,17Z))	HMDB0013442	
PC.ae.C40.5	PC(o-20:1(11Z)/20:4(8Z,11Z,14Z,17Z))	HMDB0013444	
PC.ae.C40.6	PC(o-18:0/22:6(4Z,7Z,10Z,13Z,16Z,19Z))	HMDB0013422	

PC.ae.C42.1	PC(o-18:1(9Z)/24:0)	HMDB0013434	
PC.ae.C42.2	PC(o-18:2(9Z,12Z)/24:0)	HMDB0013438	
PC.ae.C42.3	PC(o-24:0/18:3(6Z,9Z,12Z))	HMDB0013458	
PC.ae.C42.4	PC(o-22:0/20:4(8Z,11Z,14Z,17Z))	HMDB0013448	
PC.ae.C44.3	PC(o-22:0/22:3(10Z,13Z,16Z))	HMDB0013449	
PC.ae.C44.4	PC(o-22:1(13Z)/22:3(10Z,13Z,16Z))	HMDB0013453	
PC.ae.C44.5	PC(o-22:2(13Z,16Z)/22:3(10Z,13Z,16Z))	HMDB0013456	
PC.ae.C44.6	PC(o-22:0/22:6(4Z,7Z,10Z,13Z,16Z,19Z))	HMDB0013450	
SM..OH..C14.1	SM(d18:0/14:1(9Z)(OH))	HMDB0013462	C00550
SM..OH..C16.1	SM OH C16.1	HMDB0013463	C00550
SM..OH..C22.1	SM(d18:0/22:1(13Z)(OH))	HMDB0013466	C00550
SM..OH..C22.2	SM(d18:0/22:2(13Z,16Z)(OH))	HMDB0013467	C00550
SM..OH..C24.1	SM(d18:0/24:1(15Z)(OH))	HMDB0013469	C00550
SM.C16.0	SM(d18:1/16:0)	HMDB0010169	C00550
SM.C16.1	SM C16:1	HMDB0029216	
SM.C18.0	SM(d18:1/18:0)	HMDB0001348	C00550
SM.C18.1	SM(d18:1/18:1(9Z))	HMDB0012101	C00550
SM.C20.2	SM(d18:0/20:2(11Z,14Z))	HMDB0013465	C00550
SM.C22.3	SM(d18:0/22:3(10Z,13Z,16Z))	HMDB0013468	C00550
SM.C24.0	SM(d18:1/24:0)	HMDB0011697	
SM.C24.1	SM(d18:1/24:1(15Z))	HMDB0012107	C00550
SM.C26.0	SM(d18:1/26:0)	HMDB0011698	C00550
SM.C26.1	SM(d18:0/26:1(17Z))	HMDB0013461	C00550
H1	D-Glucose	HMDB0000122	C00031
Lactic.acid	L-Lactic acid	HMDB0000190	C00256
beta.Hydroxybutyric.acid	3-Hydroxybutyric acid	HMDB0000357	C01089
alpha.Ketoglutaric.acid	Oxoglutaric acid	HMDB0000208	C00026
Citric.acid	Citric acid	HMDB0000094	C00158
Butyric.acid	Butyric acid	HMDB0000039	C00246
Propionic.acid	Propionic acid	HMDB0000237	C00163
HPHPA	3-(3-Hydroxyphenyl)-3-hydroxypropanoic acid	HMDB0002643	
Succinic.acid	Succinic acid	HMDB0000254	C00042
Fumaric.acid	Fumaric acid	HMDB0000134	C00122
Pyruvic.acid	Pyruvic acid	HMDB0000243	C00022
Isobutyric.acid	Isobutyric acid	HMDB0001873	C02632
Hippuric.acid	Hippuric acid	HMDB0000714	C01586
Methylmalonic.acid	Methylmalonic acid	HMDB0000202	C02170
Homovanillic.acid	Homovanillic acid	HMDB0000118	C05582

Indole.acetic.acid	Indoleacetic acid	HMDB0000197	C00954
Uric.acid	Uric acid	HMDB0000289	C00366
5-Hydroxyindole.acetic.acid	5-Hydroxyindoleacetic acid	HMDB0000763	C05635
p-Hydroxyphenylacetic.acid	p-Hydroxyphenylacetic acid	HMDB0000020	C00642
ratio_AA	Total aromatic amino acids (Tyrosine, Tryptophane, Phenylalanine, and Histidine)		
ratio_BCAA	Total branched amino acids (Isoleucine, Leucine, and Valine)		
ratio_Fischer	Fischer's ratio (Isoleucine, Leucine, and Valine / Tyrosine and Phenylalanine)		
ratio_Essential_AA	Total essential amino acids (Phenylalanine, Valine, Threonine, Tryptophan, Methionine, Leucine, Isoleucine, Lysine, and Histidine)		
ratio_Glucogenic_AA	Total glucogenic amino acids (Alanine, Arginine, Asparagine, Aspartic, Glutamic, Glutamine, Glycine, Histidine, Methionine, Proline, Serine, and Valine)		
ratio_Keto_AA	Total ketogenic amino acids (Leucine and Lysine)		
ratio_Total_AAs	Total amino acids		
ratio_urea_cycle	Total urea cycle amino acids (Citrulline, Ornitine, Argining, Aspartic acid)		
ratio_Kynurenine_Trp	Kynurenine to Trptophan ratio		
ratio_Orn_Arg	Ornithine to Arginine ratio		
ratio_Orn_Putrescine	Ornithine to Putrescine ratio		
ratio_Serotonin_Trp	Serotonin to Trptophan ratio		
ratio_Spermidine_Putrescine	Spermidine to Putrescine ratio		
ratio_Spermine_Spermidine	Spermine to Spermidine ratio		
ratio_Tyr_Phe	Tyrosine to Phenylalanine ratio		
ratio_Cit_Orn	Citrulline to Ornithine ratio		
ratio_Cit_Arg	Citrulline to Arginine ratio		
ratio_C2_C0	Acetylcarnitine to carnitine ratio		

Table S2. Metabolomics univariate results.

**Metabolite**	**coef**	**se.coef**	**OR**	**95CI.OR**	**P**	**P.FDR**	**mean_control**	**mean_case**	**fold_change**
Ala	-1.48	0.71	0.23	[0.0565 to 0.916]	0.04	0.10	297.71	246.31	0.83
Arg	-2.16	0.79	0.12	[0.0246 to 0.536]	0.01	0.03	82.02	67.52	0.82
Asn	0.86	0.61	2.37	[0.723 to 7.75]	0.15	0.25	53.69	61.22	1.14
Asp	-0.91	0.58	0.41	[0.131 to 1.25]	0.12	0.21	38.27	34.28	0.90
Cit	-0.55	0.37	0.58	[0.283 to 1.19]	0.14	0.22	11.21	7.68	0.68
Gln	-0.01	0.76	0.99	[0.223 to 4.37]	0.99	0.99	493.25	504.52	1.02
Glu	-0.17	0.47	0.84	[0.335 to 2.11]	0.71	0.79	138.13	164.46	1.19
Gly	-0.02	0.78	0.98	[0.214 to 4.48]	0.98	0.99	319.44	325.88	1.02
His	2.41	1.01	11.20	[1.56 to 80.3]	0.02	0.06	95.20	111.84	1.17

Ile	-1.72	0.70	0.18	[0.045 to 0.711]	0.02	0.06	60.25	48.71	0.81
Leu	-1.54	0.77	0.21	[0.0478 to 0.959]	0.04	0.12	118.86	100.06	0.84
Lys	-1.45	0.89	0.23	[0.0408 to 1.34]	0.10	0.20	189.42	166.42	0.88
Met	-0.94	0.61	0.39	[0.118 to 1.3]	0.13	0.21	20.43	18.26	0.89
Orn	-1.07	0.54	0.34	[0.118 to 0.995]	0.05	0.13	63.49	51.28	0.81
Phe	1.12	0.81	3.05	[0.618 to 15]	0.17	0.26	116.55	131.07	1.12
Pro	-1.31	0.85	0.27	[0.0513 to 1.42]	0.12	0.21	217.47	200.59	0.92
Ser	-1.80	0.85	0.17	[0.0315 to 0.865]	0.03	0.10	189.32	169.64	0.90
Thr	-0.21	0.69	0.81	[0.209 to 3.12]	0.76	0.82	81.39	78.26	0.96
Trp	-0.87	0.40	0.42	[0.192 to 0.907]	0.03	0.09	26.24	19.43	0.74
Tyr	0.00	0.54	1.00	[0.349 to 2.89]	0.99	0.99	55.23	53.24	0.96
Val	-1.46	0.77	0.23	[0.0512 to 1.05]	0.06	0.14	179.45	152.91	0.85
alpha AAA	0.59	0.46	1.80	[0.739 to 4.4]	0.20	0.28	1.28	1.53	1.19
Creatinine	2.59	0.79	13.30	[2.84 to 62]	0.00	0.01	26.90	39.85	1.48
Kynurenine	-0.15	0.34	0.86	[0.445 to 1.66]	0.65	0.73	2.51	2.51	1.00
Putrescine	0.68	0.34	1.97	[1.02 to 3.82]	0.04	0.12	0.21	0.28	1.33
Serotonin	-0.60	0.25	0.55	[0.337 to 0.895]	0.02	0.06	0.84	0.65	0.77
Spermidine	0.33	0.58	1.39	[0.45 to 4.32]	0.56	0.66	0.53	0.56	1.06
Spermine	0.71	0.51	2.04	[0.746 to 5.58]	0.16	0.26	0.34	0.36	1.08
t4.OH.Pro	1.66	0.68	5.25	[1.4 to 19.7]	0.01	0.06	15.03	19.17	1.28
Taurine	-1.20	0.64	0.30	[0.0856 to 1.05]	0.06	0.14	130.78	116.97	0.89
C0 Acylcarnitine	-1.19	0.68	0.30	[0.0805 to 1.14]	0.08	0.16	20.41	18.82	0.92
C2 Acylcarnitine	1.80	0.58	6.07	[1.93 to 19.1]	0.00	0.01	14.70	20.50	1.39
C3 Acylcarnitine	0.47	0.53	1.60	[0.565 to 4.5]	0.38	0.47	0.41	0.48	1.17
C4-OH Acylcarnitine	0.89	0.45	2.43	[1 to 5.89]	0.05	0.13	0.26	0.34	1.31
C4 Acylcarnitine	1.82	0.73	6.20	[1.5 to 25.7]	0.01	0.05	0.19	0.22	1.19
lysoPC a C16.0	-2.67	0.59	0.07	[0.0218 to 0.22]	0.00	0.00	34.40	19.55	0.57
lysoPC a C16.1	-2.58	0.60	0.08	[0.0233 to 0.246]	0.00	0.00	0.70	0.48	0.69
lysoPC a C17.0	-2.86	0.69	0.06	[0.0148 to 0.22]	0.00	0.00	0.39	0.23	0.60
lysoPC a C18.0	-2.83	0.63	0.06	[0.0173 to 0.203]	0.00	0.00	8.85	4.70	0.53
lysoPC a C18.1	-2.87	0.62	0.06	[0.0169 to 0.192]	0.00	0.00	7.93	4.70	0.59
lysoPC a C18.2	-2.19	0.49	0.11	[0.0426 to 0.293]	0.00	0.00	8.97	4.91	0.55
lysoPC a C20.3	-2.72	0.59	0.07	[0.0209 to 0.209]	0.00	0.00	0.54	0.31	0.57
lysoPC a C20.4	-2.24	0.54	0.11	[0.0372 to 0.306]	0.00	0.00	2.45	1.57	0.64
lysoPC a C24.0	-1.16	0.63	0.31	[0.0908 to 1.09]	0.07	0.15	0.18	0.14	0.76
PC aa C24.0	0.52	0.59	1.68	[0.534 to 5.31]	0.37	0.47	0.18	0.18	0.98
PC aa C28.1	-0.61	0.66	0.55	[0.15 to 1.98]	0.36	0.46	1.40	1.30	0.93
PC aa C30.0	0.67	0.59	1.96	[0.623 to 6.18]	0.25	0.35	3.77	4.38	1.16
PC aa C32.0	1.95	0.75	7.01	[1.6 to 30.7]	0.01	0.04	16.98	20.53	1.21
PC aa C32.1	1.46	0.52	4.30	[1.56 to 11.8]	0.00	0.03	13.71	19.67	1.43
PC aa C32.2	1.28	0.60	3.59	[1.11 to 11.6]	0.03	0.10	1.41	1.75	1.24
PC aa C32.3	-1.21	0.91	0.30	[0.0503 to 1.77]	0.18	0.27	0.14	0.13	0.91

PC aa C34.1	1.45	0.81	4.28	[0.884 to 20.7]	0.07	0.15	198.47	228.50	1.15
PC aa C34.2	0.30	0.89	1.34	[0.237 to 7.62]	0.74	0.81	236.79	239.21	1.01
PC aa C34.3	0.06	0.59	1.07	[0.339 to 3.35]	0.91	0.94	5.00	4.81	0.96
PC aa C34.4	-0.70	0.61	0.50	[0.149 to 1.66]	0.25	0.36	0.33	0.29	0.90
PC aa C36.0	-0.91	0.67	0.40	[0.108 to 1.51]	0.18	0.27	1.76	1.29	0.73
PC aa C36.1	0.38	0.69	1.47	[0.382 to 5.62]	0.58	0.67	34.94	37.88	1.08
PC aa C36.2	-0.12	0.74	0.89	[0.207 to 3.81]	0.87	0.91	126.90	125.80	0.99
PC aa C36.3	-1.36	0.79	0.26	[0.0543 to 1.2]	0.08	0.17	44.86	38.50	0.86
PC aa C36.4	-0.34	0.76	0.71	[0.162 to 3.14]	0.66	0.73	90.09	85.63	0.95
PC aa C36.5	-0.91	0.54	0.40	[0.141 to 1.16]	0.09	0.18	6.03	4.79	0.79
PC aa C36.6	-1.11	0.60	0.33	[0.101 to 1.08]	0.07	0.15	0.23	0.19	0.84
PC aa C38.0	-0.96	0.63	0.39	[0.111 to 1.33]	0.13	0.22	1.93	1.57	0.81
PC aa C38.3	-2.06	0.86	0.13	[0.0237 to 0.689]	0.02	0.06	16.78	13.91	0.83
PC aa C38.4	-0.08	0.76	0.92	[0.206 to 4.12]	0.92	0.94	45.56	44.34	0.97
PC aa C38.5	-1.56	0.83	0.21	[0.0415 to 1.06]	0.06	0.14	17.89	15.59	0.87
PC aa C38.6	-1.17	0.52	0.31	[0.112 to 0.852]	0.02	0.08	47.66	34.75	0.73
PC aa C40.2	-0.46	0.88	0.63	[0.114 to 3.52]	0.60	0.69	0.15	0.13	0.87
PC aa C40.3	-1.45	0.92	0.23	[0.0385 to 1.43]	0.12	0.21	0.25	0.22	0.87
PC aa C40.4	-1.77	0.84	0.17	[0.0327 to 0.885]	0.04	0.10	1.50	1.32	0.87
PC aa C40.5	-2.00	0.79	0.14	[0.0285 to 0.638]	0.01	0.05	4.20	3.53	0.84
PC aa C40.6	-1.34	0.57	0.26	[0.0864 to 0.794]	0.02	0.06	16.18	12.14	0.75
PC aa C42.0	-1.01	0.69	0.37	[0.0942 to 1.41]	0.14	0.23	0.30	0.25	0.83
PC aa C42.1	-1.68	0.74	0.19	[0.0436 to 0.792]	0.02	0.08	0.16	0.13	0.79
PC aa C42.2	-1.03	0.80	0.36	[0.0754 to 1.7]	0.20	0.28	0.11	0.10	0.85
PC aa C42.4	-2.07	0.89	0.13	[0.0223 to 0.721]	0.02	0.07	0.09	0.08	0.83
PC aa C42.5	-1.87	0.89	0.16	[0.0268 to 0.891]	0.04	0.10	0.16	0.13	0.85
PC ae C30.0	0.13	0.53	1.13	[0.406 to 3.17]	0.81	0.86	0.27	0.29	1.07
PC ae C32.1	1.51	0.83	4.51	[0.883 to 23]	0.07	0.15	2.46	2.75	1.12
PC ae C32.2	1.63	0.90	5.09	[0.871 to 29.8]	0.07	0.15	0.35	0.40	1.12
PC ae C34.0	1.11	0.79	3.03	[0.652 to 14.1]	0.16	0.25	0.82	0.90	1.09
PC ae C34.1	1.30	0.85	3.66	[0.697 to 19.2]	0.13	0.21	6.01	6.61	1.10
PC ae C34.2	-0.65	0.99	0.52	[0.0758 to 3.61]	0.51	0.61	4.32	4.10	0.95
PC ae C34.3	-1.35	0.84	0.26	[0.0503 to 1.34]	0.11	0.20	2.65	2.29	0.86
PC ae C36.0	0.29	0.71	1.34	[0.333 to 5.37]	0.68	0.76	0.68	0.68	1.01
PC ae C36.1	0.10	0.76	1.11	[0.252 to 4.88]	0.89	0.92	3.40	3.40	1.00
PC ae C36.2	-0.42	0.88	0.66	[0.119 to 3.65]	0.63	0.72	4.85	4.61	0.95
PC ae C36.3	-1.52	0.99	0.22	[0.0316 to 1.5]	0.12	0.21	2.57	2.31	0.90
PC ae C36.4	-0.79	0.80	0.45	[0.0949 to 2.17]	0.32	0.43	9.61	8.81	0.92
PC ae C36.5	-0.14	0.76	0.87	[0.195 to 3.89]	0.86	0.90	6.33	6.09	0.96
PC ae C38.0	-2.33	0.83	0.10	[0.0192 to 0.488]	0.00	0.03	0.66	0.51	0.77
PC ae C38.1	1.09	0.48	2.98	[1.16 to 7.62]	0.02	0.08	0.36	0.36	1.01
PC ae C38.2	-1.78	0.83	0.17	[0.0331 to 0.866]	0.03	0.10	0.80	0.64	0.81

PC ae C38.3	-2.17	0.88	0.11	[0.0204 to 0.633]	0.01	0.05	1.15	0.96	0.83
PC ae C38.4	-0.82	0.89	0.44	[0.0762 to 2.53]	0.36	0.46	6.41	6.03	0.94
PC ae C38.5	-0.67	0.85	0.51	[0.0961 to 2.72]	0.43	0.53	8.75	8.25	0.94
PC ae C38.6	-0.59	0.67	0.55	[0.149 to 2.05]	0.38	0.47	3.60	3.14	0.87
PC ae C40.1	-2.29	0.84	0.10	[0.0198 to 0.524]	0.01	0.03	0.34	0.26	0.76
PC ae C40.2	-2.85	0.89	0.06	[0.00999 to 0.333]	0.00	0.01	0.53	0.40	0.76
PC ae C40.3	-1.56	0.96	0.21	[0.0323 to 1.36]	0.10	0.20	0.36	0.32	0.88
PC ae C40.4	-1.36	0.91	0.26	[0.0432 to 1.53]	0.14	0.22	1.07	0.97	0.91
PC ae C40.5	-1.36	0.85	0.26	[0.0489 to 1.34]	0.11	0.20	1.52	1.37	0.90
PC ae C40.6	-1.23	0.73	0.29	[0.0702 to 1.21]	0.09	0.18	2.37	1.99	0.84
PC ae C42.1	-0.49	0.68	0.62	[0.163 to 2.32]	0.47	0.57	0.16	0.14	0.91
PC ae C42.2	-2.65	0.82	0.07	[0.0143 to 0.348]	0.00	0.01	0.20	0.15	0.73
PC ae C42.3	-2.64	0.88	0.07	[0.0127 to 0.405]	0.00	0.02	0.25	0.19	0.77
PC ae C42.4	-1.01	0.54	0.37	[0.126 to 1.06]	0.06	0.14	0.38	0.32	0.83
PC ae C44.3	-0.66	0.60	0.52	[0.161 to 1.66]	0.27	0.37	0.06	0.05	0.87
PC ae C44.4	-1.23	0.64	0.29	[0.0833 to 1.02]	0.05	0.13	0.16	0.13	0.84
PC ae C44.5	-0.59	0.62	0.55	[0.163 to 1.87]	0.34	0.45	0.68	0.63	0.93
PC ae C44.6	-0.69	0.71	0.50	[0.125 to 1.99]	0.33	0.43	0.64	0.59	0.91
SM .OH..C14.1	-0.98	0.75	0.38	[0.0867 to 1.63]	0.19	0.28	2.04	1.92	0.94
SM .OH..C16.1	-1.44	0.90	0.24	[0.041 to 1.37]	0.11	0.20	1.33	1.22	0.91
SM .OH..C22.1	-2.99	0.81	0.05	[0.0104 to 0.245]	0.00	0.00	4.14	2.79	0.68
SM .OH..C22.2	-2.19	0.79	0.11	[0.0238 to 0.527]	0.01	0.03	3.05	2.43	0.80
SM .OH..C24.1	-2.39	0.83	0.09	[0.0181 to 0.46]	0.00	0.02	0.48	0.37	0.76
SM C16.0	-0.18	0.89	0.84	[0.146 to 4.77]	0.84	0.89	86.95	84.94	0.98
SM C16.1	0.81	0.79	2.24	[0.481 to 10.5]	0.30	0.41	8.33	9.14	1.10
SM C18.0	-1.31	0.82	0.27	[0.0545 to 1.34]	0.11	0.20	17.32	15.40	0.89
SM C18.1	-0.53	0.79	0.59	[0.126 to 2.74]	0.50	0.60	5.76	5.48	0.95
SM C20.2	-0.61	0.83	0.55	[0.108 to 2.74]	0.46	0.56	0.21	0.20	0.94
SM C24.0	-3.44	0.92	0.03	[0.0053 to 0.193]	0.00	0.00	12.34	8.69	0.70
SM C24.1	-1.40	0.72	0.25	[0.0596 to 1.01]	0.05	0.13	40.25	33.16	0.82
SM C26.0	-3.13	0.86	0.04	[0.00809 to 0.237]	0.00	0.00	0.07	0.05	0.69
SM C26.1	-3.26	0.87	0.04	[0.00695 to 0.213]	0.00	0.00	0.17	0.12	0.71
H1	-0.67	0.61	0.51	[0.155 to 1.69]	0.27	0.37	4609.68	4689.85	1.02
Lactic acid	1.13	0.81	3.09	[0.629 to 15.2]	0.16	0.26	3129.11	3408.76	1.09
beta-Hydroxybutyric acid	0.48	0.25	1.61	[0.984 to 2.63]	0.06	0.14	664.00	894.15	1.35
alpha-Ketoglutaric acid	0.95	0.51	2.58	[0.953 to 6.97]	0.06	0.14	12.12	16.92	1.40

Citric acid	0.75	0.56	2.11	[0.706 to 6.32]	0.18	0.27	17.43	21.51	1.23
Butyric acid	4.55	1.15	94.40	[10 to 891]	0.00	0.00	1.62	2.25	1.39
Propionic acid	7.93	1.63	2770.00	[113 to 67800]	0.00	0.00	2.41	3.51	1.45
HPHPA	-0.58	0.46	0.56	[0.226 to 1.39]	0.21	0.30	0.03	0.02	0.68
Succinic acid	1.02	0.55	2.77	[0.952 to 8.06]	0.06	0.14	4.27	4.60	1.08
Fumaric acid	2.47	0.68	11.80	[3.11 to 44.7]	0.00	0.00	2.18	3.45	1.58
Pyruvic acid	1.99	0.72	7.30	[1.8 to 29.6]	0.01	0.03	113.62	144.46	1.27
Isobutyric acid	7.59	1.63	1980.00	[81.7 to 47800]	0.00	0.00	0.92	1.63	1.76
Hippuric acid	-0.05	0.16	0.95	[0.689 to 1.31]	0.75	0.81	1.39	1.99	1.43
Methylmalonic acid	0.74	0.44	2.10	[0.884 to 4.97]	0.09	0.18	0.18	0.29	1.61
Homovanillic acid	3.38	0.72	29.30	[7.18 to 120]	0.00	0.00	0.04	0.15	3.96
Indole.acetic acid	0.02	0.30	1.02	[0.573 to 1.82]	0.94	0.96	1.05	0.91	0.87
Uric acid	2.40	0.75	11.00	[2.54 to 47.6]	0.00	0.01	523.53	745.33	1.42
5-Hydroxyindole.acetic acid	0.77	0.37	2.16	[1.05 to 4.46]	0.04	0.10	0.07	0.11	1.56
p-Hydroxyphenylacetic acid	0.15	0.24	1.17	[0.731 to 1.86]	0.52	0.61	1.92	2.12	1.10
ratio_AAA	0.00	0.00	1.00	[0.999 to 1.01]	0.12	0.21	293.23	315.58	1.08
ratio_BCAA	0.00	0.00	1.00	[0.995 to 1]	0.02	0.06	358.56	301.69	0.84
ratio_Fischer	-0.79	0.27	0.45	[0.27 to 0.761]	0.00	0.02	2.10	1.75	0.83
ratio_Essential_AA	0.00	0.00	1.00	[0.998 to 1]	0.17	0.26	887.79	826.96	0.93
ratio_Glucogenic_AA	0.00	0.00	1.00	[0.999 to 1]	0.53	0.62	2124.39	2057.43	0.97
ratio_Keto_AA	0.00	0.00	1.00	[0.994 to 1]	0.03	0.08	308.28	266.48	0.86
ratio_Kynurenine_Trp	4.16	1.56	64.30	[3.04 to 1360]	0.01	0.04	0.12	0.17	1.43
ratio_Orn_Arg	0.28	0.21	1.32	[0.88 to 1.98]	0.18	0.27	0.80	0.98	1.23
ratio_Orn_Putrescine	0.00	0.00	1.00	[0.999 to 1]	0.08	0.16	823.35	481.63	0.58
ratio_Serotonin_Trp	1.13	2.18	3.11	[0.0436 to 221]	0.60	0.69	0.05	0.05	1.11
ratio_Spermidine_Putrescine	-0.01	0.01	0.99	[0.965 to 1.01]	0.38	0.47	9.16	4.55	0.50
ratio_Spermine_Spermidine	0.68	0.44	1.98	[0.829 to 4.71]	0.12	0.21	0.72	0.81	1.11
ratio_Tyr_Phe	-0.55	0.56	0.58	[0.192 to 1.73]	0.33	0.43	0.48	0.44	0.92
ratio_Cit_Orn	-0.39	0.44	0.68	[0.288 to 1.59]	0.37	0.47	0.26	0.21	0.80
ratio_Cit_Arg	-0.24	0.90	0.79	[0.136 to 4.54]	0.79	0.84	0.15	0.14	0.95
ratio_C2_C0	0.69	0.20	2.00	[1.34 to 2.98]	0.00	0.01	0.87	1.39	1.60
ratio_Total_AAs	0.00	0.00	1.00	[1 to 1]	0.33	0.43	2847.02	2713.58	0.95
ratio_urea_cycle	-0.01	0.00	0.99	[0.989 to 0.998]	0.01	0.03	194.98	160.75	0.82

Table S3. Proteomic univariate results.

Protein	coef	se.coef	OR	95CI.OR	P	P.FDR	mean_control	mean_case	fold_change
A0A024CIM4	-0.581	0.367	0.559	[0.272 to 1.15]	0.11	0.8	9.08	8.97	1.01
A0A024R035	-0.0758	0.29	0.927	[0.525 to 1.64]	0.79	0.88	10.65	10.63	1.00
A0A024R2Q7	0.112	0.376	1.12	[0.535 to 2.33]	0.77	0.88	8.01	8.03	1.00
A0A024R3E3	-0.35	0.291	0.705	[0.398 to 1.25]	0.23	0.8	12.71	12.61	1.01
A0A024R462	0.315	0.333	1.37	[0.714 to 2.63]	0.34	0.8	9.37	9.43	0.99
A0A024R6I6	-0.0783	0.382	0.925	[0.437 to 1.96]	0.84	0.9	9.08	9.07	1.00
A0A024R6I7	0.489	0.339	1.63	[0.839 to 3.17]	0.15	0.8	13.34	13.44	0.99
A0A024R6I9	-0.477	0.367	0.621	[0.302 to 1.28]	0.19	0.8	10.20	10.11	1.01
A0A024R6P0	0.522	0.357	1.69	[0.837 to 3.4]	0.14	0.8	12.75	12.85	0.99
A0A024R944	-0.91	0.401	0.402	[0.184 to 0.882]	0.023	0.8	11.72	11.57	1.01
A0A024RAA7	-0.105	0.413	0.901	[0.401 to 2.02]	0.8	0.88	9.32	9.31	1.00
A0A024RAG6	-0.141	0.389	0.868	[0.405 to 1.86]	0.72	0.87	8.69	8.67	1.00
A0A044QNR9	0.722	0.338	2.06	[1.06 to 4]	0.033	0.8	9.31	9.47	0.98
A0A087X1J7	-0.105	0.291	0.9	[0.509 to 1.59]	0.72	0.87	9.39	9.36	1.00
A0A096LPE2	-0.236	0.279	0.79	[0.457 to 1.37]	0.4	0.8	9.26	9.19	1.01
A0A0A0MRJ7	-0.112	0.407	0.894	[0.403 to 1.99]	0.78	0.88	10.09	10.08	1.00
A0A0A0MS15	0.184	0.233	1.2	[0.762 to 1.9]	0.43	0.8	8.74	8.82	0.99
A0A0A0MSV6	0.164	0.436	1.18	[0.501 to 2.77]	0.71	0.87	9.27	9.29	1.00
A0A0B4J1V2	0.321	0.218	1.38	[0.899 to 2.11]	0.14	0.8	7.68	7.85	0.98
A0A0C4DH31	0.479	0.302	1.61	[0.893 to 2.92]	0.11	0.8	7.87	7.99	0.98
A0A0C4DH33	0.226	0.34	1.25	[0.644 to 2.44]	0.51	0.8	7.82	7.87	0.99
A0A0C4DH67	0.403	0.351	1.5	[0.752 to 2.98]	0.25	0.8	9.82	9.89	0.99
A0A0F7SYK7	0.401	0.289	1.49	[0.848 to 2.63]	0.17	0.8	8.34	8.45	0.99
A0A0F7T7M3	0.208	0.26	1.23	[0.739 to 2.05]	0.42	0.8	8.00	8.07	0.99
A0A0G2JMY9	0.0663	0.37	1.07	[0.517 to 2.21]	0.86	0.91	7.81	7.82	1.00
A0A0S2Z3D5	0.606	0.36	1.83	[0.905 to 3.71]	0.092	0.8	10.85	10.96	0.99
A0A0S2Z3Y1	0.381	0.365	1.46	[0.717 to 2.99]	0.3	0.8	10.65	10.71	0.99
A0A0U1RR20	-0.147	0.336	0.863	[0.447 to 1.67]	0.66	0.87	8.25	8.22	1.00
A0A0X9UWK7	0.253	0.226	1.29	[0.828 to 2]	0.26	0.8	8.86	8.97	0.99
A0A0X9UWL5	0.256	0.262	1.29	[0.772 to 2.16]	0.33	0.8	9.51	9.59	0.99
A0A109PSY4	0.205	0.265	1.23	[0.731 to 2.06]	0.44	0.8	8.11	8.18	0.99
A0A125QYY9	0.467	0.308	1.6	[0.872 to 2.92]	0.13	0.8	8.84	8.96	0.99
A0A140VJI7	-0.289	0.429	0.749	[0.323 to 1.74]	0.5	0.8	9.59	9.55	1.00
A0A140VJJ6	0.287	0.271	1.33	[0.784 to 2.26]	0.29	0.8	11.78	11.87	0.99
A0A140VK00	-0.0819	0.377	0.921	[0.44 to 1.93]	0.83	0.9	11.23	11.22	1.00
A0A140VK24	-0.306	0.353	0.737	[0.369 to 1.47]	0.39	0.8	8.37	8.32	1.01
A0A140VKF3	0.695	0.434	2	[0.857 to 4.69]	0.11	0.8	10.92	11.01	0.99
A0A193CHQ9	0.278	0.255	1.32	[0.801 to 2.18]	0.28	0.8	8.85	8.95	0.99
A0A1S5UZ39	0.124	0.235	1.13	[0.715 to 1.79]	0.6	0.84	11.11	11.16	1.00
A0A1U9X793	-0.118	0.343	0.889	[0.454 to 1.74]	0.73	0.87	8.84	8.82	1.00
A0A1W6IYJ6	0.477	0.36	1.61	[0.796 to 3.26]	0.19	0.8	8.55	8.64	0.99
A0A1Z1VXF2	-0.0177	0.318	0.982	[0.527 to 1.83]	0.96	0.97	8.23	8.23	1.00
A0A286YES1	0.2	0.236	1.22	[0.77 to 1.94]	0.4	0.8	10.19	10.28	0.99
A0A2Q2TTZ9	0.294	0.262	1.34	[0.803 to 2.24]	0.26	0.8	8.74	8.84	0.99
A0A2U8J8D9	0.463	0.295	1.59	[0.89 to 2.83]	0.12	0.8	9.62	9.75	0.99
A0A2U8J8Q6	0.241	0.222	1.27	[0.823 to 1.97]	0.28	0.8	7.93	8.04	0.99

A0A2Z4LCH4	-0.287	0.4	0.75	[0.343 to 1.64]	0.47	0.8	10.60	10.56	1.00
A0A346D5N4	-0.277	0.345	0.758	[0.385 to 1.49]	0.42	0.8	8.94	8.88	1.01
A0A5E4	0.266	0.239	1.31	[0.818 to 2.08]	0.26	0.8	9.04	9.15	0.99
A0M8Q7	0.115	0.25	1.12	[0.687 to 1.83]	0.65	0.87	7.73	7.77	0.99
A0N071	0.0671	0.277	1.07	[0.622 to 1.84]	0.81	0.88	9.98	10.00	1.00
A0N5G1	0.24	0.222	1.27	[0.823 to 1.96]	0.28	0.8	9.02	9.14	0.99
A0N5G5	0.16	0.194	1.17	[0.802 to 1.72]	0.41	0.8	7.56	7.65	0.99
A2J1N5	0.31	0.286	1.36	[0.777 to 2.39]	0.28	0.8	8.23	8.32	0.99
A2KBB9	0.282	0.262	1.33	[0.792 to 2.22]	0.28	0.8	9.52	9.62	0.99
A2MYD4	0.178	0.203	1.19	[0.802 to 1.78]	0.38	0.8	7.81	7.91	0.99
A2N0T4	0.244	0.242	1.28	[0.794 to 2.05]	0.31	0.8	8.07	8.17	0.99
A2NB45	0.247	0.36	1.28	[0.632 to 2.59]	0.49	0.8	7.99	8.03	0.99
A2NUT2	0.25	0.223	1.28	[0.829 to 1.99]	0.26	0.8	8.22	8.34	0.99
A2NYV4	0.345	0.254	1.41	[0.857 to 2.32]	0.18	0.8	8.44	8.56	0.99
A2RTY6	-0.311	0.38	0.733	[0.348 to 1.54]	0.41	0.8	11.54	11.49	1.00
A5PL27	-0.343	0.354	0.71	[0.355 to 1.42]	0.33	0.8	13.00	12.94	1.00
A5YAK2	-0.241	0.293	0.786	[0.443 to 1.39]	0.41	0.8	7.51	7.45	1.01
A8K008	0.185	0.197	1.2	[0.818 to 1.77]	0.35	0.8	11.95	12.07	0.99
A8K5J8	-0.14	0.385	0.869	[0.409 to 1.85]	0.71	0.87	10.82	10.80	1.00
A8K5T0	-0.292	0.38	0.746	[0.355 to 1.57]	0.44	0.8	11.48	11.44	1.00
B0YIW2	0.109	0.262	1.11	[0.667 to 1.86]	0.68	0.87	10.02	10.06	1.00
B2R4C5	0.0409	0.36	1.04	[0.515 to 2.11]	0.91	0.93	8.39	8.39	1.00
B2R582	-0.219	0.393	0.803	[0.372 to 1.74]	0.58	0.83	9.37	9.33	1.00
B2R5G8	-0.209	0.362	0.811	[0.399 to 1.65]	0.56	0.82	8.79	8.75	1.00
B2R6W1	-0.346	0.293	0.707	[0.399 to 1.26]	0.24	0.8	8.23	8.13	1.01
B2R888	0.314	0.384	1.37	[0.646 to 2.9]	0.41	0.8	8.56	8.61	0.99
B2R8I2	-0.94	0.354	0.391	[0.195 to 0.781]	0.0079	0.46	NA	NA	NA
B4DF70	-0.134	0.306	0.874	[0.48 to 1.59]	0.66	0.87	9.56	9.53	1.00
B4DPP8	-0.278	0.389	0.757	[0.353 to 1.62]	0.47	0.8	8.41	8.36	1.01
B4DQA0	-0.279	0.363	0.757	[0.372 to 1.54]	0.44	0.8	8.97	8.92	1.01
B4E0X1	0.259	0.32	1.3	[0.692 to 2.43]	0.42	0.8	9.29	9.34	0.99
B4E1C2	-0.438	0.351	0.645	[0.324 to 1.28]	0.21	0.8	11.81	11.73	1.01
B4E1D8	-0.687	0.422	0.503	[0.22 to 1.15]	0.1	0.8	11.52	11.42	1.01
B4E1Z4	-0.284	0.419	0.753	[0.331 to 1.71]	0.5	0.8	12.33	12.29	1.00
B7Z539	-0.309	0.271	0.735	[0.432 to 1.25]	0.25	0.8	9.05	8.95	1.01
B7Z550	-0.667	0.453	0.513	[0.211 to 1.25]	0.14	0.8	10.30	10.22	1.01
B7Z8Q2	-0.0878	0.347	0.916	[0.464 to 1.81]	0.8	0.88	11.37	11.36	1.00
B7ZKJ8	0.153	0.402	1.17	[0.529 to 2.56]	0.7	0.87	12.26	12.28	1.00
C0JYY2	-0.0931	0.374	0.911	[0.438 to 1.9]	0.8	0.88	13.70	13.68	1.00
C9JF17	-0.385	0.336	0.68	[0.352 to 1.31]	0.25	0.8	9.39	9.31	1.01
CON__ENSEMBL.ENSBTAP00000024466	0.125	0.303	1.13	[0.625 to 2.05]	0.68	0.87	9.06	9.09	1.00
CON__P00761	0.0448	0.54	1.05	[0.363 to 3.01]	0.93	0.95	8.61	8.62	1.00
CON__P02768.1	0.0557	0.216	1.06	[0.692 to 1.62]	0.8	0.88	12.97	13.00	1.00
D3DQX7	0.205	0.2	1.23	[0.83 to 1.82]	0.3	0.8	9.72	9.84	0.99
D3GKD8	0.242	0.243	1.27	[0.791 to 2.05]	0.32	0.8	9.36	9.45	0.99
D3JV41	-0.169	0.249	0.845	[0.518 to 1.38]	0.5	0.8	9.39	9.33	1.01
D9IWP9	-0.371	0.312	0.69	[0.374 to 1.27]	0.23	0.8	10.79	10.70	1.01
D9ZGG2	0.0727	0.424	1.08	[0.468 to 2.47]	0.86	0.92	11.07	11.08	1.00

E9KL23	0.134	0.25	1.14	[0.701 to 1.87]	0.59	0.84	9.72	9.77	0.99
E9KL26	0.2	0.399	1.22	[0.559 to 2.67]	0.62	0.86	11.86	11.89	1.00
E9KL36	-0.242	0.39	0.785	[0.365 to 1.69]	0.53	0.81	11.03	11.00	1.00
E9PFZ2	-0.0782	0.186	0.925	[0.642 to 1.33]	0.67	0.87	9.44	9.39	1.01
G3XAM2	-0.105	0.429	0.901	[0.388 to 2.09]	0.81	0.88	10.66	10.65	1.00
H0YAC1	-0.454	0.388	0.635	[0.297 to 1.36]	0.24	0.8	10.63	10.56	1.01
I3L145	-0.31	0.372	0.733	[0.354 to 1.52]	0.4	0.8	10.01	9.96	1.01
J3QQR8	0.714	0.415	2.04	[0.905 to 4.61]	0.085	0.8	8.43	8.54	0.99
K7ER74	0.0952	0.277	1.1	[0.639 to 1.89]	0.73	0.87	9.91	9.94	1.00
K7ERI9	-0.0294	0.254	0.971	[0.59 to 1.6]	0.91	0.93	9.36	9.35	1.00
L7UYB8	0.709	0.377	2.03	[0.97 to 4.26]	0.06	0.8	9.13	9.26	0.99
O00391	-0.176	0.418	0.839	[0.369 to 1.9]	0.67	0.87	8.54	8.51	1.00
O14791	-0.243	0.284	0.784	[0.45 to 1.37]	0.39	0.8	9.73	9.66	1.01
O43866	-0.211	0.315	0.81	[0.437 to 1.5]	0.5	0.8	9.26	9.21	1.01
O75636	0.375	0.394	1.46	[0.673 to 3.15]	0.34	0.8	10.18	10.23	0.99
O75882	-0.0566	0.434	0.945	[0.403 to 2.21]	0.9	0.93	10.05	10.04	1.00
P00734	-0.294	0.401	0.745	[0.339 to 1.64]	0.46	0.8	11.83	11.78	1.00
P00738	-0.197	0.176	0.822	[0.582 to 1.16]	0.26	0.8	12.54	12.39	1.01
P00739	-0.106	0.288	0.899	[0.511 to 1.58]	0.71	0.87	10.16	10.13	1.00
P00747	-0.472	0.328	0.624	[0.328 to 1.19]	0.15	0.8	11.35	11.24	1.01
P01023	-0.0826	0.285	0.921	[0.526 to 1.61]	0.77	0.88	13.93	13.90	1.00
P01031	-0.0411	0.404	0.96	[0.435 to 2.12]	0.92	0.94	12.04	12.03	1.00
P01591	-0.0822	0.268	0.921	[0.545 to 1.56]	0.76	0.88	8.94	8.92	1.00
P01742	0.224	0.264	1.25	[0.745 to 2.1]	0.4	0.8	8.16	8.23	0.99
P01861	0.309	0.252	1.36	[0.831 to 2.23]	0.22	0.8	8.83	8.95	0.99
P01871	0.124	0.227	1.13	[0.726 to 1.76]	0.59	0.83	11.61	11.67	1.00
P02671	0.269	0.264	1.31	[0.781 to 2.19]	0.31	0.8	12.11	12.20	0.99
P02741	0.575	0.206	1.78	[1.19 to 2.66]	0.0052	0.4	10.01	10.38	0.96
P02750	0.409	0.351	1.51	[0.757 to 2.99]	0.24	0.8	11.16	11.24	0.99
P02760	-0.286	0.399	0.751	[0.344 to 1.64]	0.47	0.8	10.97	10.92	1.00
P02774	0.00861	0.334	1.01	[0.524 to 1.94]	0.98	0.98	9.46	9.46	1.00
P02790	-0.221	0.301	0.802	[0.445 to 1.45]	0.46	0.8	12.29	12.24	1.00
P03951	-0.312	0.491	0.732	[0.28 to 1.92]	0.53	0.8	8.57	8.54	1.00
P04040	0.264	0.345	1.3	[0.662 to 2.56]	0.45	0.8	9.23	9.28	0.99
P04070	-0.375	0.434	0.687	[0.294 to 1.61]	0.39	0.8	8.26	8.22	1.01
P04275	0.941	0.372	2.56	[1.24 to 5.31]	0.011	0.53	10.07	10.25	0.98
P04430	0.233	0.275	1.26	[0.736 to 2.16]	0.4	0.8	8.73	8.80	0.99
P04433	0.177	0.228	1.19	[0.764 to 1.87]	0.44	0.8	8.50	8.58	0.99
P05109	0.743	0.254	2.1	[1.28 to 3.46]	0.0034	0.4	8.74	9.08	0.96
P05543	-0.128	0.364	0.88	[0.431 to 1.8]	0.72	0.87	10.61	10.59	1.00
P05546	-0.6	0.293	0.549	[0.309 to 0.974]	0.04	0.8	10.46	10.28	1.02
P06312	0.122	0.211	1.13	[0.747 to 1.71]	0.56	0.82	7.73	7.79	0.99
P06331	0.144	0.195	1.16	[0.788 to 1.69]	0.46	0.8	8.47	8.56	0.99
P06396	-0.189	0.394	0.828	[0.382 to 1.79]	0.63	0.87	11.16	11.14	1.00
P06681	-0.0804	0.414	0.923	[0.41 to 2.08]	0.85	0.9	9.47	9.46	1.00
P06702	0.732	0.256	2.08	[1.26 to 3.44]	0.0043	0.4	9.93	10.26	0.97
P06727	0.435	0.315	1.54	[0.834 to 2.86]	0.17	0.8	11.77	11.87	0.99
P07195	0.385	0.428	1.47	[0.634 to 3.4]	0.37	0.8	9.17	9.21	0.99
P07225	-0.597	0.457	0.551	[0.225 to 1.35]	0.19	0.8	9.58	9.51	1.01
P07357	-0.635	0.402	0.53	[0.241 to 1.17]	0.11	0.8	9.83	9.73	1.01

P07360	-0.554	0.457	0.575	[0.235 to 1.41]	0.23	0.8	9.42	9.36	1.01
P07996	-0.284	0.313	0.753	[0.407 to 1.39]	0.37	0.8	9.95	9.88	1.01
P08697	0.0201	0.388	1.02	[0.477 to 2.18]	0.96	0.97	11.24	11.24	1.00
P09871	-0.158	0.404	0.854	[0.387 to 1.88]	0.7	0.87	10.53	10.51	1.00
P0C0L4	0.218	0.329	1.24	[0.653 to 2.37]	0.51	0.8	10.29	10.34	1.00
P0C0L5	0.148	0.371	1.16	[0.56 to 2.4]	0.69	0.87	13.11	13.14	1.00
P0DOX2	0.437	0.256	1.55	[0.937 to 2.56]	0.088	0.8	8.69	8.85	0.98
P0DOX3	0.0926	0.248	1.1	[0.675 to 1.78]	0.71	0.87	9.69	9.72	1.00
P0DOX7	0.117	0.239	1.12	[0.703 to 1.8]	0.63	0.87	9.16	9.21	0.99
P0DP02	0.395	0.323	1.48	[0.788 to 2.8]	0.22	0.8	7.53	7.62	0.99
P0DP08	0.0434	0.313	1.04	[0.566 to 1.93]	0.89	0.93	8.28	8.29	1.00
P0DP09	0.136	0.214	1.15	[0.753 to 1.74]	0.53	0.8	8.03	8.10	0.99
P10643	0.352	0.405	1.42	[0.642 to 3.15]	0.39	0.8	11.49	11.54	1.00
P10909	-0.253	0.344	0.777	[0.396 to 1.52]	0.46	0.8	10.60	10.55	1.00
P13671	-0.581	0.369	0.56	[0.271 to 1.15]	0.12	0.8	10.89	10.79	1.01
P15169	-0.13	0.442	0.878	[0.369 to 2.09]	0.77	0.88	10.04	10.03	1.00
P18065	0.188	0.31	1.21	[0.657 to 2.22]	0.54	0.81	8.51	8.56	0.99
P19652	0.4	0.295	1.49	[0.837 to 2.66]	0.18	0.8	11.01	11.12	0.99
P19827	-0.424	0.378	0.655	[0.312 to 1.37]	0.26	0.8	11.39	11.31	1.01
P20851	-0.869	0.421	0.419	[0.184 to 0.956]	0.039	0.8	9.81	9.69	1.01
P22792	0.333	0.446	1.4	[0.582 to 3.35]	0.46	0.8	10.51	10.54	1.00
P23142	-0.135	0.417	0.874	[0.386 to 1.98]	0.75	0.88	8.49	8.48	1.00
P27169	-0.109	0.312	0.897	[0.487 to 1.65]	0.73	0.87	10.32	10.29	1.00
P35858	-0.0574	0.385	0.944	[0.444 to 2.01]	0.88	0.92	8.54	8.53	1.00
P36980	-0.237	0.383	0.789	[0.373 to 1.67]	0.54	0.81	8.54	8.53	1.00
P43251	-0.126	0.422	0.882	[0.385 to 2.02]	0.77	0.88	9.21	9.19	1.00
P43652	-0.0571	0.371	0.944	[0.456 to 1.96]	0.88	0.92	10.92	10.91	1.00
P51884	0.0573	0.389	1.06	[0.494 to 2.27]	0.88	0.92	10.68	10.69	1.00
P68871	0.0721	0.24	1.07	[0.672 to 1.72]	0.76	0.88	11.96	11.99	1.00
Q04756	-0.161	0.373	0.852	[0.41 to 1.77]	0.67	0.87	9.19	9.17	1.00
Q06033	0.501	0.435	1.65	[0.703 to 3.88]	0.25	0.8	10.76	10.82	0.99
Q0KKI6	0.234	0.186	1.26	[0.877 to 1.82]	0.21	0.8	7.97	8.13	0.98
Q0ZCG4	0.112	0.271	1.12	[0.658 to 1.9]	0.68	0.87	7.60	7.63	1.00
Q0ZCH9	0.334	0.255	1.4	[0.847 to 2.3]	0.19	0.8	9.03	9.15	0.99
Q1KLZ0	0.36	0.288	1.43	[0.815 to 2.52]	0.21	0.8	10.51	10.61	0.99
Q53GN8	-0.617	0.388	0.54	[0.252 to 1.15]	0.11	0.8	8.77	8.67	1.01
Q53GU8	0.487	0.419	1.63	[0.716 to 3.7]	0.25	0.8	9.04	9.11	0.99
Q53H26	-0.0847	0.198	0.919	[0.624 to 1.35]	0.67	0.87	12.15	12.10	1.00
Q5CAQ5	0.197	0.335	1.22	[0.631 to 2.35]	0.56	0.82	8.32	8.36	1.00
Q5JVE7	-0.581	0.395	0.559	[0.258 to 1.21]	0.14	0.8	9.22	9.13	1.01
Q5NV88	0.36	0.275	1.43	[0.836 to 2.46]	0.19	0.8	7.77	7.88	0.99
Q5NV90	0.222	0.233	1.25	[0.791 to 1.97]	0.34	0.8	7.92	8.02	0.99
Q5NV92	0.148	0.284	1.16	[0.664 to 2.02]	0.6	0.84	7.82	7.87	0.99
Q5T619	0.433	0.288	1.54	[0.876 to 2.71]	0.13	0.8	7.80	7.93	0.98
Q5VY30	-0.344	0.335	0.709	[0.368 to 1.37]	0.3	0.8	10.55	10.48	1.01
Q6GMX0	0.131	0.406	1.14	[0.515 to 2.53]	0.75	0.88	7.56	7.57	1.00
Q6GMX6	0.323	0.26	1.38	[0.829 to 2.3]	0.21	0.8	8.97	9.08	0.99
Q6LAM1	-0.429	0.404	0.651	[0.295 to 1.44]	0.29	0.8	8.51	8.45	1.01
Q6N089	0.249	0.282	1.28	[0.738 to 2.23]	0.38	0.8	8.49	8.56	0.99
Q6N093	0.131	0.221	1.14	[0.739 to 1.76]	0.55	0.82	9.99	10.06	0.99

Q6NS95	0.198	0.214	1.22	[0.801 to 1.86]	0.35	0.8	9.53	9.63	0.99
Q6PIL8	0.22	0.203	1.25	[0.837 to 1.85]	0.28	0.8	11.48	11.60	0.99
Q86TT1	0.361	0.246	1.44	[0.887 to 2.32]	0.14	0.8	8.42	8.57	0.98
Q86U78	0.818	0.372	2.27	[1.09 to 4.7]	0.028	0.8	11.60	11.76	0.99
Q8N355	0.225	0.201	1.25	[0.845 to 1.86]	0.26	0.8	11.35	11.48	0.99
Q8N5F4	0.291	0.219	1.34	[0.87 to 2.06]	0.18	0.8	8.47	8.62	0.98
Q8TCF0	0.431	0.329	1.54	[0.808 to 2.93]	0.19	0.8	9.13	9.23	0.99
Q96K68	0.22	0.234	1.25	[0.787 to 1.97]	0.35	0.8	10.67	10.76	0.99
Q96PD5	-0.37	0.35	0.691	[0.348 to 1.37]	0.29	0.8	10.35	10.28	1.01
Q96SA9	0.203	0.236	1.23	[0.772 to 1.95]	0.39	0.8	9.64	9.73	0.99
Q9NPP6	0.137	0.192	1.15	[0.787 to 1.67]	0.48	0.8	7.75	7.84	0.99
Q9NSD6	0.111	0.236	1.12	[0.704 to 1.78]	0.64	0.87	8.11	8.16	0.99
Q9NZP8	-0.173	0.393	0.841	[0.39 to 1.82]	0.66	0.87	9.06	9.03	1.00
Q9UL70	0.448	0.286	1.57	[0.894 to 2.74]	0.12	0.8	8.27	8.40	0.98
Q9UL78	0.141	0.228	1.15	[0.736 to 1.8]	0.54	0.81	8.50	8.56	0.99
Q9UL83	0.217	0.233	1.24	[0.786 to 1.96]	0.35	0.8	8.76	8.85	0.99
Q9UL90	0.108	0.195	1.11	[0.76 to 1.63]	0.58	0.83	8.78	8.84	0.99
Q9UPN9	0.147	0.23	1.16	[0.738 to 1.82]	0.52	0.8	8.20	8.27	0.99
S6AWF4	0.136	0.205	1.15	[0.767 to 1.71]	0.51	0.8	7.69	7.77	0.99
S6BAM6	0.198	0.229	1.22	[0.779 to 1.91]	0.39	0.8	7.99	8.08	0.99
S6BGD6	0.311	0.218	1.37	[0.89 to 2.09]	0.15	0.8	7.93	8.09	0.98
S6BGE0	0.129	0.195	1.14	[0.777 to 1.67]	0.51	0.8	8.29	8.37	0.99
S6BGF5	0.127	0.179	1.14	[0.799 to 1.61]	0.48	0.8	7.70	7.79	0.99
V9GYM3	-0.468	0.272	0.626	[0.367 to 1.07]	0.086	0.8	11.08	10.92	1.01
V9HVY1	0.215	0.261	1.24	[0.744 to 2.07]	0.41	0.8	12.14	12.21	0.99
V9HVZ4	0.53	0.35	1.7	[0.856 to 3.37]	0.13	0.8	8.69	8.79	0.99
V9HW21	0.0771	0.269	1.08	[0.637 to 1.83]	0.77	0.88	8.66	8.68	1.00
V9HW68	0.123	0.188	1.13	[0.783 to 1.63]	0.51	0.8	8.90	8.98	0.99
V9HWA9	-0.473	0.383	0.623	[0.294 to 1.32]	0.22	0.8	13.89	13.81	1.01
V9HWD8	-0.183	0.408	0.833	[0.374 to 1.85]	0.65	0.87	11.51	11.49	1.00
V9HWE3	-0.109	0.302	0.897	[0.496 to 1.62]	0.72	0.87	10.08	10.05	1.00
V9HWF6	0.552	0.302	1.74	[0.961 to 3.14]	0.068	0.8	12.37	12.52	0.99
V9HWI1	0.277	0.398	1.32	[0.604 to 2.88]	0.49	0.8	7.94	7.98	0.99
V9HWI6	-0.0739	0.361	0.929	[0.458 to 1.88]	0.84	0.9	12.48	12.47	1.00
V9HWJ7	0.799	0.416	2.22	[0.984 to 5.03]	0.055	0.8	9.08	9.19	0.99
V9HWN7	0.447	0.38	1.56	[0.742 to 3.29]	0.24	0.8	8.93	9.01	0.99
V9HWP0	-0.00349	0.277	0.997	[0.579 to 1.71]	0.99	0.99	9.83	9.83	1.00

Table S4. Healthy non-stunted community subjects.

**Anthropometric Features**	**Health community (HC) subjects**
Age (months), mean± sd	37.1 ± 13.3
MUAC (cm), mean± sd	15.2 ± 1.6
WHZ, mean± sd	0.4 ± 2.3
WAZ, mean± sd	-0.5 ± 1.7
HAZ, mean± sd	-1.3 ± 0.7
BMIZ, mean± sd	-0.2 ± 0.0

MUAC = mid-upper arm circumference, WHZ = weight for height z score, WAZ = weight for age z score, HAZ = height for age z score, BMIZ = body mass index z score, sd = standard deviation.

Table S5. Cytokines and chemokines measured.

Protein Alias	Name	Official Symbol
EGF	epidermal growth factor	EGF
Eotaxin	chemokine (C-C motif) ligand 11	CCL11
GCSF	colony stimulating factor 3 (granulocyte)	CSF3
GMCSF	colony stimulating factor 2 (granulocyte-macrophage)	CSF2
IFNα2	interferon, alpha 2	IFNA2
IFNγ	interferon, gamma	IFNG
IL10	interleukin 10	IL10
IL12p40	interleukin 12B (40kDa subunit of IL12)	IL12B
IL12p70	interleukin 12 (active heterodimer of IL12A and IL12B)	IL12A and B
IL13	interleukin 13	IL13
IL15	interleukin 15	IL15
IL17A	interleukin 17A	IL17A
IL1RA	interleukin 1 receptor antagonist	IL1RN
IL1α	interleukin 1, alpha	IL1A
IL1β	interleukin 1, beta	IL1B
IL2	interleukin 2	IL2
IL3	interleukin 3	IL3
IL4	interleukin 4	IL4
IL5	interleukin 5	IL5
IL6	interleukin 6	IL6
IL7	interleukin 7	IL7
IL8	interleukin 8	IL8
IP10	chemokine (C-X-C motif) ligand 10	IP10
MCP1	chemokine (C-C motif) ligand 2	CCL2
MIP1α	chemokine (C-C motif) ligand 3	CCL3
MIP1β	chemokine (C-C motif) ligand 4	CCL4
TNFα	tumor necrosis factor	TNF
TNFβ	lymphotoxin alpha	LTA
VEGF	vascular endothelial growth factor	VEGF

REFERENCES AND NOTES

1. R. E. Black, C. G. Victora, S. P. Walker, Z. A. Bhutta, P. Christian, M. de Onis, M. Ezzati, S. Grantham-McGregor, J. Katz, R. Martorell, R. Uauy, Maternal and child undernutrition and overweight in low-income and middle-income countries. *Lancet* **382**, 427–451 (2013).

2. Guideline: Updates on the Management of Severe Acute Malnutrition in Infants and Children. WHO. World Health Organization, Geneva (2013).

3. M. Hossain, M. J. Chisti, M. I. Hossain, M. Mahfuz, M. M. Islam, T. Ahmed, Efficacy of World Health Organization guideline in facility-based reduction of mortality in severely malnourished children from low and middle income countries: A systematic review and meta-analysis. *J. Paediatr. Child Health* **53**, 474–479 (2017).

4. S. Bartz, A. Mody, C. Hornik, J. Bain, M. Muehlbauer, T. Kiyimba, E. Kiboneka, R. Stevens, J. Bartlett, J. V. St Peter, C. B. Newgard, M. Freemark, Severe acute malnutrition in childhood: Hormonal and metabolic status at presentation, response to treatment, and predictors of mortality. *J. Clin. Endocrinol. Metab.* **99**, 2128–2137 (2014).

5. V. Di Giovanni, C. Bourdon, D. X. Wang, S. Seshadri, E. Senga, C. J. Versloot, W. Voskuijl, R. D. Semba, I. Trehan, R. Moaddel, M. I. Ordiz, L. Zhang, J. Parkinson, M. J. Manary, R. H. J. Bandsma, Metabolomic changes in serum of children with different clinical diagnoses of malnutrition. *J. Nutr.* **146**, 2436–2444 (2016).

6. A. McMillan, A. E. Orimadegun, M. W. Sumarah, J. Renaud, M. M. da Encarnacao, G. B. Gloor, O. O. Akinyinka, G. Reid, S. J. Allen, Metabolic derangements identified through untargeted metabolomics in a cross-sectional study of Nigerian children with severe acute malnutrition. *Metabolomics* **13**, (2017).

7. R. H. J. Bandsma, M. Mendel, M. N. Spoelstra, D. J. Reijngoud, T. Boer, F. Stellaard, B. Brabin, R. Schellekens, E. Senga, G. T. Heikens, Mechanisms behind decreased endogenous glucose production in malnourished children. *Pediatr. Res.* **68**, 423–428 (2010).

8. F. Jahoor, A. Badaloo, M. Reid, T. Forrester, Protein metabolism in severe childhood malnutrition.

Ann. Trop. Paediatr. **28**, 87–101 (2008).

9. A. V. Badaloo, T. Forrester, M. Reid, F. Jahoor, Lipid kinetic differences between children with kwashiorkor and those with marasmus. *Am. J. Clin. Nutr.* **83**, 1283–1288 (2006).

10. J. C. Waterlow, Oxidative phosphorylation in livers of normal and malnourished human infants. *Proc. R. Soc. Ser. B* **155**, 96–114 (1961).

11. T. van Zutphen, J. Ciapaite, V. W. Bloks, C. Ackereley, A. Gerding, A. Jurdzinski, R. A. de Moraes, L. Zhang, J. C. Wolters, R. Bischoff, R. J. Wanders, S. M. Houten, D. Bronte-Tinkew, T. Shatseva, G. F. Lewis, A. K. Groen, D. J. Reijngoud, B. M. Bakker, J. W. Jonker, P. K. Kim, R. H. J. Bandsma, Malnutrition-associated liver steatosis and ATP depletion is caused by peroxisomal and mitochondrial dysfunction. *J. Hepatol.* **65**, 1198–1208 (2016).

12. S. Attia, C. J. Versloot, W. Voskuijl, S. J. van Vliet, V. Di Giovanni, L. Zhang, S. Richardson, C. Bourdon, M. G. Netea, J. A. Berkley, P. F. van Rheenen, R. H. Bandsma, Mortality in children with complicated severe acute malnutrition is related to intestinal and systemic inflammation: An observational cohort study. *Am. J. Clin. Nutr.* **104**, 1441–1449 (2016).

13. M. Singer, V. De Santis, D. Vitale, W. Jeffcoate, Multiorgan failure is an adaptive, endocrine-mediated, metabolic response to overwhelming systemic inflammation. *Lancet* **364**, 545–548 (2004).

14. J. Wang, Y. Sun, S. Teng, K. Li, Prediction of sepsis mortality using metabolite biomarkers in the blood: A meta-analysis of death-related pathways and prospective validation. *BMC Med.* **18**, 83 (2020).

15. R. Steuer, Review: On the analysis and interpretation of correlations in metabolomic data. *Brief. Bioinform.* **7**, 151–158 (2006).

16. K. Sharma, K. M. Mogensen, M. K. Robinson, Pathophysiology of critical illness and role of nutrition. *Nutr. Clin. Pract.* **34**, 12–22 (2019).

17. Z. A. Bhutta, J. A. Berkley, R. H. J. Bandsma, M. Kerac, I. Trehan, A. Briend, Severe childhood malnutrition. *Nat. Rev. Dis. Primers* **3**, 17067 (2017).

18. S. E. H. Brooks, J. F. Doherty, M. H. N. Golden, Peroxisomes and the hepatic pathology of childhood malnutrition. *West Indian Med. J.* **43**, 15–17 (1994).

19. M. G. Schooneman, F. M. Vaz, S. M. Houten, M. R. Soeters, Acylcarnitines reflecting or inflicting insulin resistance? *Diabetes* **62**, 1–8 (2013).

20. M. Baggiolini, A. Walz, S. L. Kunkel, Neutrophil-activating peptide-1/interleukin 8, a novel cytokine that activates neutrophils. *J. Clin. Invest.* **84**, 1045–1049 (1989).

21. R. L. Guerrant, A. M. Leite, R. Pinkerton, P. H. Medeiros, P. A. Cavalcante, M. DeBoer, M. Kosek, C. Duggan, A. Gewirtz, J. C. Kagan, A. E. Gauthier, J. Swann, J. Mayneris-Perxachs, D. T. Bolick, E. A. Maier, M. M. Guedes, S. R. Moore, W. A. Petri, A. Havt, I. F. Lima, M. M. Prata, J. C. Michaleckyj, R. J. Scharf, C. Sturgeon, A. Fasano, A. A. Lima, Biomarkers of environmental enteropathy, inflammation, stunting, and impaired growth in children in Northeast Brazil. *PLOS ONE* **11**, e0158772 (2016).

22. S. Attia, M. Feenstra, N. Swain, M. Cuesta, R. H. J. Bandsma, Starved guts: Morphologic and functional intestinal changes in malnutrition. *J. Pediatr. Gastroenterol. Nutr.* **65**, 491–495 (2017).

23. F. Haussner, S. Chakraborty, R. Halbgebauer, M. Huber-Lang, Challenge to the intestinal mucosa during sepsis. *Front. Immunol.* **10**, 891 (2019).

24. J. Weng, H. Wu, Z. Xu, H. T. Xi, C. Chen, D. Q. Chen, Y. Q. Gong, Y. Hua, Z. Y. Wang, The role of propionic acid at diagnosis predicts mortality in patients with septic shock. *J. Crit. Care* **43**, 95–101 (2018).

25. B. Mickiewicz, H. J. Vogel, H. R. Wong, B. W. Winston, Metabolomics as a novel approach for early diagnosis of pediatric septic shock and its mortality. *Am. J. Respir. Crit. Care Med.* **187**, 967–976 (2013).

26. Z. A. Ktsoyan, M. S. Mkrtchyan, M. K. Zakharyan, A. A. Mnatsakanyan, K. A. Arakelova, Z. U. Gevorgyan, A. M. Sedrakyan, A. I. Hovhannisyan, A. A. Arakelyan, R. I. Aminov, Systemic concentrations of short chain fatty acids are elevated in salmonellosis and exacerbation of familial Mediterranean fever. *Front. Microbiol.* **7**, (2016).

27. L. Van Wyngene, J. Vandewalle, C. Libert, Reprogramming of basic metabolic pathways in microbial sepsis: Therapeutic targets at last? *EMBO Mol. Med.* **10**, (2018).

28. R. D. Semba, M. Shardell, I. Trehan, R. Moaddel, K. M. Maleta, M. I. Ordiz, K. Kraemer, M. Khadeer, L. Ferrucci, M. J. Manary, Metabolic alterations in children with environmental enteric dysfunction. *Sci. Rep.* **6**, (2016).

29. J. H. Kabarowski, K. Zhu, L. Q. Le, O. N. Witte, Y. Xu, Lysophosphatidylcholine as a ligand for the immunoregulatory receptor G2A. *Science (New York, N.Y.)* **293**, 702–705 (2001).

30. S. D. Wright, R. N. Kolesnick, Does endotoxin stimulate cells by mimicking ceramide? *Immunol. Today* **16**, 297–302 (1995).

31. A. Cambiaghi, B. B. Pinto, L. Brunelli, F. Falcetta, F. Aletti, K. Bendjelid, R. Pastorelli, M. Ferrario, Characterization of a metabolomic profile associated with responsiveness to therapy in the acute phase of septic shock. *Sci. Rep.* **7**, 9748 (2017).

32. W. Drobnik, G. Liebisch, F. X. Audebert, D. Frohlich, T. Gluck, P. Vogel, G. Rothe, G. Schmitz, Plasma ceramide and lysophosphatidylcholine inversely correlate with mortality in sepsis patients. *J. Lipid Res.* **44**, 754–761 (2003).

33. D. C. Muller, A. Kauppi, A. Edin, A. Gylfe, A. B. Sjostedt, A. Johansson, Phospholipid levels in blood during community-acquired pneumonia. *PLOS ONE* **14**, e0216379 (2019).

34. J. J. Yan, J. S. Jung, J. E. Lee, J. Lee, S. O. Huh, H. S. Kim, K. C. Jung, J. Y. Cho, J. S. Nam, H. W. Suh, Y. H. Kim, D. K. Song, Therapeutic effects of lysophosphatidylcholine in experimental sepsis. *Nat. Med.* **10**, 161–167 (2004).

35. H. B. Suliman, K. E. Welty-Wolf, M. S. Carraway, D. A. Schwartz, J. W. Hollingsworth, C. A. Piantadosi, Toll-like receptor 4 mediates mitochondrial DNA damage and biogenic responses after heat-inactivated E. coli. *FASEB J.* **19**, 1531–1533 (2005).

36. A. P. West, I. E. Brodsky, C. Rahner, D. K. Woo, H. Erdjument-Bromage, P. Tempst, M. C. Walsh, Y. Choi, G. S. Shadel, S. Ghosh, TLR signalling augments macrophage bactericidal activity through

mitochondrial ROS. *Nature* **472**, 476–480 (2011).

37. J. E. Carre, J.-C. Orban, L. Re, K. Felsmann, W. Iffert, M. Bauer, H. B. Suliman, C. A. Piantadosi, T. M. Mayhew, P. Breen, M. Stotz, M. Singer, Survival in critical illness is associated with early activation of mitochondrial biogenesis. *Am. J. Respir. Crit. Care Med.* **182**, 745–751 (2010).

38. I. Giovannini, G. Boldrini, M. Castagneto, G. Sganga, G. Nanni, M. Pittiruti, G. Castiglioni, Respiratory quotient and patterns of substrate utilization in human sepsis and trauma. *J. Parenter. Enter. Nutr.* **7**, 226–230 (1983).

39. R. J. Langley, E. L. Tsalik, J. C. van Velkinburgh, S. W. Glickman, B. J. Rice, C. P. Wang, B. Chen, L. Carin, A. Suarez, R. P. Mohney, D. H. Freeman, M. Wang, J. You, J. Wulff, J. W. Thompson, M. A. Moseley, S. Reisinger, B. T. Edmonds, B. Grinnell, D. R. Nelson, D. L. Dinwiddie, N. A. Miller, C. J. Saunders, S. S. Soden, A. J. Rogers, L. Gazourian, L. E. Fredenburgh, A. F. Massaro, R. M. Baron, A. M. K. Choi, G. R. Corey, G. S. Ginsburg, C. B. Cairns, R. M. Otero, V. G. Fowler, E. P. Rivers, C. W. Woods, S. F. Kingsmore, An integrated clinico-metabolomic model improves prediction of death in sepsis. *Sci. Transl. Med.* **5**, 195ra95 (2013).

40. F. Amin, M. Davidson, K. L. Davis, Homovanillic-acid measurement in clinical research—A review of methodology. *Schizophr. Bull.* **18**, 123–148 (1992).

41. F. Valerio, P. Lavermicocca, M. Pascale, A. Visconti, Production of phenyllactic acid by lactic acid bacteria: An approach to the selection of strains contributing to food quality and preservation. *FEMS Microbiol. Lett.* **233**, 289–295 (2004).

42. N. V. Beloborodova, Y. N. Sarshor, A. Y. Bedova, E. A. Chernevskaya, A. K. Pautova, Involvement of aromatic metabolites in the pathogenesis of septic shock. *Shock* **50**, 273–279 (2018).

43. E. Chernevskaya, N. Beloborodova, N. Klimenko, A. Pautova, D. Shilkin, V. Gusarov, A. Tyakht, Serum and fecal profiles of aromatic microbial metabolites reflect gut microbiota disruption in critically ill patients: A prospective observational pilot study. *Crit. Care* **24**, 312 (2020).

44. J. L. Gehrig, S. Venkatesh, H. W. Chang, M. C. Hibberd, V. L. Kung, J. Y. Cheng, R. Y. Chen, S. Subramanian, C. A. Cowardin, M. F. Meier, D. O'Donnell, M. Talcott, L. D. Spears, C. F.

Semenkovich, B. Henrissat, R. J. Giannone, R. L. Hettich, O. Ilkayeva, M. Muehlbauer, C. B. Newgard, C. Sawyer, R. D. Head, D. A. Rodionov, A. A. Arzamasov, S. A. Leyn, A. L. Osterman, M. I. Hossain, M. Islam, N. Choudhury, S. A. Sarker, S. Huq, I. Mahmud, I. Mostafa, M. Mahfuz, M. J. Barratt, T. Ahmed, J. I. Gordon, Effects of microbiota-directed foods in gnotobiotic animals and undernourished children. *Science* **365**, eaau4732 (2019).

45. J. M. Njunge, A. Gwela, N. K. Kibinge, M. Ngari, L. Nyamako, E. Nyatichi, J. Thitiri, G. B. Gonzales, R. H. J. Bandsma, J. L. Walson, E. N. Gitau, J. A. Berkley, Biomarkers of post-discharge mortality among children with complicated severe acute malnutrition. *Sci. Rep.* **9**, 5981 (2019).

46. T. Dailey-Chwalibog, M. Freemark, M. Muehlbauer, D. Roberfroid, I. A. Kemokai, M. R. Mostak, M. A. Alim, M. Khan, M. A. Khan, L. Bawo, N. K. Dunbar, C. H. Taylor, H. Fouillet, J. F. Huneau, P. Kolsteren, B. Guesdon, Clinical and biochemical markers of risk in uncomplicated severe acute malnutrition. *Pediatrics* **147**, e2020027003 (2021).

47. E. van Puffelen, J. M. Hulst, I. Vanhorebeek, K. Duffer, G. Van den Berghe, S. C. A. T. Verbruggen, K. F. M. Joosten, Outcomes of delaying parenteral nutrition for 1 week vs initiation within 24 hours among undernourished children in pediatric intensive care: A subanalysis of the PEPaNIC randomized clinical trial. *JAMA Netw. Open* **1**, e182668 (2018).

48. R. H. J. Bandsma, W. Voskuijl, E. Chimwezi, G. Fegan, A. Briend, J. Thitiri, M. Ngari, L. Mwalekwa, V. Bandika, R. Ali, F. Hamid, B. Owor, N. Mturi, I. Potani, B. Allubha, A. C. M. Kobold, R. H. Bartels, C. J. Versloot, M. Feenstra, D. A. van den Brink, P. F. van Rheenen, M. Kerac, C. Bourdon, J. A. Berkley, A reduced-carbohydrate and lactose-free formulation for stabilization among hospitalized children with severe acute malnutrition: A double-blind, randomized controlled trial. *PLoS Med.* **16**, e1002747 (2019).

49. P. C. Austin, An introduction to propensity score methods for reducing the effects of confounding in observational studies. *Multivar. Behav. Res.* **46**, 399–424 (2011).

50. M. Kuhn, Classification and regression training, *Comprehensive R Archive Network* (2018).

51. J. Cox, M. Mann, MaxQuant enables high peptide identification rates, individualized p.p.b.-range

mass accuracies and proteome-wide protein quantification. *Nat. Biotechnol.* **26**, 1367–1372 (2008).

52. W. E. Johnson, C. Li, A. Rabinovic, Adjusting batch effects in microarray expression data using empirical Bayes methods. *Biostatistics* **8**, 118–127 (2007).

53. J. H. Friedman, T. Hastie, R. Tibshirani, Regularization paths for generalized linear models via coordinate descent. **33**, 1–22 (2010).

54. S. V. Abram, N. E. Helwig, C. A. Moodie, C. G. DeYoung, A. W. MacDonald III, N. G. Waller, Bootstrap enhanced penalized regression for variable selection with neuroimaging data. *Front. Neurosci.* **10**, 344 (2016).

55. F. Rohart, B. Gautier, A. Singh, K.-A. Le Cao, mixOmics: An R package for 'omics feature selection and multiple data integration. *PLoS Comput. Biol.* **13**, e1005752 (2017).

56. E. Szymanska, E. Saccenti, A. K. Smilde, J. A. Westerhuis, Double-check: Validation of diagnostic statistics for PLS-DA models in metabolomics studies. *Metabolomics* **8**, S3-S16 (2012).

57. J. Xia, D. S. Wishart, Using metaboanalyst 3.0 for comprehensive metabolomics data analysis. *Curr. Protoc. Bioinformatics* **55**, 14.10.11–14.10.91 (2016).

58. D. W. Huang, B. T. Sherman, R. A. Lempicki, Systematic and integrative analysis of large gene lists using DAVID bioinformatics resources. *Nat. Protoc.* **4**, 44–57 (2009).

59. A. Kamburov, R. Cavill, T. M. Ebbels, R. Herwig, H. C. Keun, Integrated pathway-level analysis of transcriptomics and metabolomics data with IMPaLA. *Bioinformatics* **27**, 2917–2918 (2011).

60. M. S. Cline, M. Smoot, E. Cerami, A. Kuchinsky, N. Landys, C. Workman, R. Christmas, I. Avila-Campilo, M. Creech, B. Gross, K. Hanspers, R. Isserlin, R. Kelley, S. Killcoyne, S. Lotia, S. Maere, J. Morris, K. Ono, V. Pavlovic, A. R. Pico, A. Vailaya, P. L. Wang, A. Adler, B. R. Conklin, L. Hood, M. Kuiper, C. Sander, I. Schmulevich, B. Schwikowski, G. J. Warner, T. Ideker, G. D. Bader, Integration of biological networks and gene expression data using Cytoscape. *Nat. Protoc.* **2**, 2366–2382 (2007).

61. J. Cox, N. Neuhauser, A. Michalski, R. A. Scheltema, J. V. Olsen, M. Mann, Andromeda: A peptide search engine integrated into the MaxQuant environment. *J. Proteome Res.* **10**, 1794–1805 (2011).

62. Y. Benjamini, Y. Hochberg, Controlling the false discovery rate: A practical and powerful approach to multiple testing. *J. R. Stat. Soc. Ser. B Stat Methodol.* **57**, 289–300 (1995).

63. D. Y. Yu, Z. Y. Zhang, K. Glass, J. Su, D. L. Demeo, K. Tantisira, S. T. Weiss, W. L. Qiu, New statistical methods for constructing robust differential correlation networks to characterize the interactions among microRNAs. *Sci. Rep.* **9**, 3499 (2019).

64. Z. M. Li, Y. X. Zhang, T. Hu, S. Likhodii, G. Sun, G. J. Zhai, Z. Z. Fan, C. J. Xuan, W. Zhang, Differential metabolomics analysis allows characterization of diversity of metabolite networks between males and females. *PLOS ONE* **13**, e0207775 (2018).

65. Z. Mousavian, A. Nowzari-Dalini, Y. Rahmatallah, A. Masoudi-Nejad, Differential network analysis and protein-protein interaction study reveals active protein modules in glucocorticoid resistance for infant acute lymphoblastic leukemia. *Mol. Med.* **25**, 36 (2019).

66. Y. Assenov, F. Ramirez, S. E. Schelhorn, T. Lengauer, M. Albrecht, Computing topological parameters of biological networks. *Bioinformatics* **24**, 282–284 (2008).

67. B. Wang, A. M. Mezlini, F. Demir, M. Fiume, Z. W. Tu, M. Brudno, B. Haibe-Kains, A. Goldenberg, Similarity network fusion for aggregating data types on a genomic scale. *Nat. Methods* **11**, 333–337 (2014).

68. N. Rappoport, R. Shamir, Multi-omic and multi-view clustering algorithms: Review and cancer benchmark. *Nucleic Acids Res.* **46**, 10546–10562 (2018).
